# Nt-acetylation-independent turnover of SQUALENE EPOXIDASE 1 by *Arabidopsis* DOA10-like E3 ligases

**DOI:** 10.1093/plphys/kiad406

**Published:** 2023-07-10

**Authors:** Ross D Etherington, Mark Bailey, Jean-Baptiste Boyer, Laura Armbruster, Xulyu Cao, Juliet C Coates, Thierry Meinnel, Markus Wirtz, Carmela Giglione, Daniel J Gibbs

**Affiliations:** School of Biosciences, University of Birmingham, Edgbaston, West Midlands, B15 2TT, UK; School of Biosciences, University of Birmingham, Edgbaston, West Midlands, B15 2TT, UK; CEA, CNRS, Université Paris-Saclay, Institute for Integrative Biology of the Cell (I2BC), Gif-sur-Yvette, 91198, France; Centre for Organismal Studies Heidelberg, Heidelberg University, Heidelberg, 69120, Germany; School of Biosciences, University of Birmingham, Edgbaston, West Midlands, B15 2TT, UK; School of Biosciences, University of Birmingham, Edgbaston, West Midlands, B15 2TT, UK; CEA, CNRS, Université Paris-Saclay, Institute for Integrative Biology of the Cell (I2BC), Gif-sur-Yvette, 91198, France; Centre for Organismal Studies Heidelberg, Heidelberg University, Heidelberg, 69120, Germany; CEA, CNRS, Université Paris-Saclay, Institute for Integrative Biology of the Cell (I2BC), Gif-sur-Yvette, 91198, France; School of Biosciences, University of Birmingham, Edgbaston, West Midlands, B15 2TT, UK

## Abstract

The acetylation-dependent (Ac/)N-degron pathway degrades proteins through recognition of their acetylated N-termini (Nt) by E3 ligases called Ac/N-recognins. To date, specific Ac/N-recognins have not been defined in plants. Here we used molecular, genetic, and multiomics approaches to characterize potential roles for *Arabidopsis* (*Arabidopsis thaliana*) DEGRADATION OF ALPHA2 10 (DOA10)-like E3 ligases in the Nt-acetylation-(NTA)-dependent turnover of proteins at global- and protein-specific scales. *Arabidopsis* has two endoplasmic reticulum (ER)-localized DOA10-like proteins. *At*DOA10A, but not the Brassicaceae-specific *At*DOA10B, can compensate for loss of yeast (*Saccharomyces cerevisiae*) *Sc*DOA10 function. Transcriptome and Nt-acetylome profiling of an *Atdoa10a/b* RNAi mutant revealed no obvious differences in the global NTA profile compared to wild type, suggesting that *At*DOA10s do not regulate the bulk turnover of NTA substrates. Using protein steady-state and cycloheximide-chase degradation assays in yeast and *Arabidopsis*, we showed that turnover of ER-localized SQUALENE EPOXIDASE 1 (*At*SQE1), a critical sterol biosynthesis enzyme, is mediated by *At*DOA10s. Degradation of *At*SQE1 *in planta* did not depend on NTA, but Nt-acetyltransferases indirectly impacted its turnover in yeast, indicating kingdom-specific differences in NTA and cellular proteostasis. Our work suggests that, in contrast to yeast and mammals, targeting of Nt-acetylated proteins is not a major function of DOA10-like E3 ligases in *Arabidopsis* and provides further insight into plant ERAD and the conservation of regulatory mechanisms controlling sterol biosynthesis in eukaryotes.

## Teaser

Global- and protein-specific analyses reveal that *Arabidopsis* DOA10A-like E3 ligases regulate the turnover of SQUALENE EPOXIDASE 1 independently of the Ac/N-degron pathway.

## Introduction

N-terminal (Nt) acetylation (NTA) is a highly prevalent chemical modification that is applied to around 60% of cytosolic proteins in yeast and more than 80% in humans and plants (Arnesen et al. 2009; Bienvenut et al. 2012; Aksnes et al. 2015). NTA is performed by N-acetyltransferase (NAT) enzymes, which catalyze the transfer of an acetyl moiety from acetyl-CoA to the α-amino group of specific Nt-residues in substrate proteins (Starheim et al. 2012). In eukaryotes, most NTA is carried out co-translationally by five ribosome-anchored NATs (NATA-NATE), with experimentally determined substrate specificities in yeast, animals, and plants (Linster et al. 2015; Aksnes et al. 2019; Huber et al. 2020). Furthermore, post-translational NTA also occurs in plants and animals via monomeric NATs that function away from the ribosome exit tunnel (Aksnes et al. 2019; Giglione and Meinnel 2021). These include membrane-bound NATF (Aksnes et al. 2015, 2017; Linster et al. 2020), a family of at least 6 plant-specific plastidic GNATs, which also catalyze lysine-acetylation (Dinh et al. 2015; Koskela et al. 2018; Bienvenut et al. 2020), and cytosolic NATH, which specifically Nt-acetylates actin in animals (Drazic et al. 2018; Wiame et al. 2018).

The addition of an acetyl group has the effect of neutralizing N-terminal charge and increasing hydrophobicity, which can influence protein fate in several ways, for example, by promoting protein–protein interactions and directing protein localization by increasing membrane affinity (Linster and Wirtz 2018; Ree et al. 2018). NTA also impacts protein folding, with deletions of NATA and particularly NATB causing the accumulation of misfolded protein aggregates in yeast (Friedrich et al. 2021). Furthermore, NTA has been shown to promote or prevent protein degradation depending on the protein target and cellular context.

In yeast and humans, acetylation of N-termini can destabilize certain proteins through the creation of Ac/N-degrons that target them for proteolysis via the acetylation-dependent (Ac/)N-degron pathway (Hwang et al. 2010; Shemorry et al. 2013; Park et al. 2015). This degradation pathway was identified in yeast (*Saccharomyces cerevisiae*), where DEGRADATION OF ALPHA2 10 (DOA10) and NEGATIVE ON TATA-LESS 4 (NOT4) E3 ligases were shown to function as Ac/N-recognins that target substrates via recognition of their Nt-acetylated N-termini (Hwang et al. 2010; Shemorry et al. 2013). Ac/N-degron pathway substrates have also been identified in humans (*Homo sapiens*) and are recognized by the human *Sc*DOA10 homolog, MEMBRANE-ASSOCIATED RING-CH-TYPE FINGER 6 (MARCH6)/TEB4 (Park et al. 2015; Nguyen et al. 2019). In *Arabidopsis* (*Arabidopsis thaliana*), NTA of an MMD-initiating isoform of SUPPRESSOR OF NPR1, CONSTITUTIVE 1 (SNC1) by NATA was shown to induce degradation, suggesting the Ac/N-degron pathway may also function in plants, though to date no plant Ac/N-recognins have been identified (Xu et al. 2015). Interestingly however, NTA of an alternative MD-initiating isoform of SNC1 by NATB was shown to have a stabilizing effect, indicating that Nt-variants of the same protein can be differentially targeted for degradation (Gibbs 2015; Xu et al. 2015). Recently, the NATA-interacting HUNTINGTIN INTERACTING PROTEIN K (HYPK) protein in rice (*Oryza sativa*) was also shown to be degraded following N-terminal acetylation (Gong et al. 2022). Since most cellular proteins are Nt-acetylated, degradation via the Ac/N-degron pathway is proposed to be conditional, with substrates only degraded when their acetylated N-termini are not internalized within a protein's structure or shielded by a binding partner (Shemorry et al. 2013).

The discovery of the Ac/N-degron pathway partially conflicted with the historical view that NTA increases protein half-life by blocking ubiquitination of the N-terminus (Hershko et al. 1984). Recent studies have also suggested that NTA does not act as a broad or universal degradation signal. High-throughput screening studies have independently shown that unstructured NTA reporter substrates of NATA and NATB were generally stable and that mutations of NATA or NATB did not increase the abundance of their endogenous targets (Kats et al. 2018; Friedrich et al. 2021). Indeed, NTA has also been reported to block other Nt-processing events such as Met-excision, arginylation, and the binding of N-recognins, thereby potentially protecting proteins from other degradative mechanisms such as the Arg/N-degron pathway (Kim et al. 2014; Park et al. 2015; Kats et al. 2018). One such protein is *Arabidopsis* SIGMA FACTOR-BINDING PROTEIN1 (SIB1), which is stabilized following NTA by NATB (Li et al. 2020). Kats et al. (2018) also observed that while mutation of *Sc*DOA10 did stabilize many normally unstable reporter proteins, turnover of these peptides was largely defined by Nt-hydrophobicity rather than NTA itself. Additionally, in human cells, NTA by NATA was shown to protect nascent proteins from degradation by preventing their unwanted interaction with IAP E3 ligases that might otherwise trigger ectopic apoptosis (Mueller et al. 2021). Moreover, an analogous proteome-wide role for NTA in protein stabilization was also recently uncovered in *Arabidopsis*, where stress-responsive NATA activity (Linster et al. 2015) was shown to mask non-Ac/N-degrons that would otherwise target NATA substrates for proteasomal degradation by as-yet-unknown E3 ligases (Gibbs et al. 2022; Linster et al. 2022).

The best characterized Ac/N-recognin, *Sc*DOA10, is a RING-type E3 ligase that localizes to the endoplasmic reticulum (ER) and nuclear envelope and which was identified as a major component of the endoplasmic reticulum-associated protein degradation (ERAD) system that degrades misfolded ER proteins (Swanson et al. 2001). *Sc*DOA10 is one of two ERAD E3 ligases and is primarily responsible for the ubiquitination of proteins with cytosolic degrons (ERAD-C) (Hirsch et al. 2009; Strasser 2018) although targeting of degrons within the ER membrane and retrotranslocase activity have also been reported for *Sc*DOA10 (Habeck et al. 2015; Schmidt et al. 2020). Two putative homologs of *Sc*DOA10 have been identified in *Arabidopsis*: *At*DOA10A, also known as ECERIFERUM9 (CER9)/SUPPRESSOR OF DRY2 DEFECTS1 (SUD1), and *At*DOA10B (Liu et al. 2011). *Atdoa10a* mutants display a range of phenotypes, including altered cuticular wax composition, improved drought tolerance, and ABA hypersensitivity during germination (Lü et al. 2012; Zhao et al. 2014). Mutations in *AtDOA10A* were also shown to repress the pleiotropic phenotypes caused by a point mutation in the sterol biosynthesis gene *SQUALENE EPOXIDASE 1* (*SQE1)/DROUGHT HYPERSENSITIVE2* (*DRY2*) by downregulating 3-HYDROY-3-METHYLGLUTARYL-COA REDUCTASE (HMGR), an upstream enzyme of the pathway (Doblas et al. 2013). It is still unclear if either *At*DOA10 homolog plays a major role in the plant ERAD system (Li et al. 2017; Huber et al. 2022). Furthermore, potential Ac/N-recognin functions for *At*DOA10s have not yet been investigated, and no physiological substrates of *At*DOA10s have been identified.

Here, we sought to functionally characterize both putative *At*DOA10 orthologs with a view to establishing whether they function as Ac/N-recognin E3 ligases of the as-yet-uncharacterized Ac/N-degron pathway in plants. Our global- and protein-specific results uncover a previously unknown function for *At*DOA10s in the homeostatic regulation of sterol biosynthesis through controlling *At*SQE1 turnover and suggest that their primary function in *Arabidopsis* is unrelated to the Ac/N-degron pathway and the bulk degradation of Nt-acetylated proteins.

## Results

### Structure, functional conservation, and phylogeny of *Arabidopsis* DOA10-like proteins

**Figure 1.**
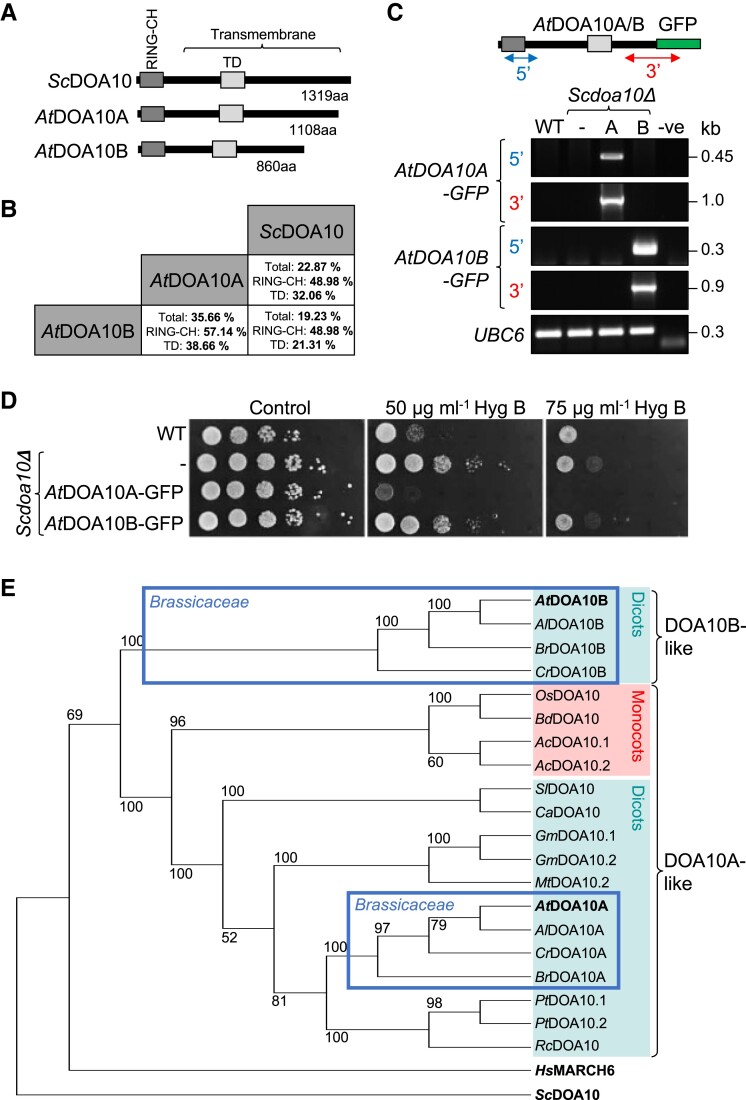
Structure, functional conservation, and phylogeny of *Arabidopsis* DOA10-like proteins. **A)** Schematic diagram of *Arabidopsis* (At) DOA10 proteins compared to the single yeast (Sc) homolog. Key domains and features are shown. **B)** Percentage identity between *Arabidopsis* and yeast DOA10 proteins. Values are shown for total protein length and the RING-CH and TD regions. See also Supplemental Fig. S1. **C)** Transgene-specific RT-PCR (using 5′ and 3′ primer pairs) confirming *AtDOA10A/B-GFP* expression in *Scdoa10Δ* mutant yeast cells. **D)** The *Scdoa10Δ* yeast mutant is insensitive to hygromycin relative to WT cells. *At*DOA10A-GFP can complement this phenotype, whereas *At*DOA10B-GFP cannot. Spots represent 10-fold dilutions from left to right. **E)** Inferred phylogenetic tree of full-length DOA10-like protein homologs identified in various diploid angiosperm species, yeast, and human (*Hs*MARCH6). Two main groups are identified: (i) a DOA10A-like clade, which is split between monocot and dicot lineages, and (ii) a DOA10B-like clade that is comprised of Brassicaceae-derived sequences only. Bootstrap values are shown, and the separate Brassicaceae groupings are highlighted with a blue box. At, *Arabidopsis thaliana*; Al, *Arabidopsis lyrata*; Br, *Brassica rapa*; Cr, *Capsella rubella*; Os, *Oryza sativa*; Bd, *Brachypodium distachyon*; Ac, *Ananas comosus*; Sl, *Solanum lycopersicum*; Ca, *Capsicum annuum*; Gm, *Glycine max*; Mt, *Medicago truncatula*; Pt, *Populus trichocarpa*; Rc, *Ricinus communis*. Tree not drawn to scale.

To identify putative DOA10 homologs in *Arabidopsis*, we searched for protein sequences with homology to full-length *Sc*DOA10 from *Saccharomyces cerevisiae*, which, in accordance with previous studies, identified two DOA10-like proteins encoded by the genes At4g34100 (*At*DOA10A/CER9/SUD1) and At4g32670 (*At*DOA10B) (Liu et al. 2011; Lü et al. 2012; Doblas et al. 2013). These share 22.87% and 19.23% amino acid identity with *Sc*DOA10, respectively, and 35.66% with each other (Fig. 1, A and B and Supplemental Fig. S1). Identity is particularly high in the N-terminal RING-CH domain, an atypical variant of the classic RING domain that provides ubiquitin ligase activity, and the TEB4/DOA10 (TD) domain, which influences interactions with the cognate ubiquitin conjugase 6 (UBC6) E2 enzyme in yeast (Fig. 1B) (Kreft and Hochstrasser 2011; Doblas et al. 2013). Both *At*DOA10s also have an extended C-terminal region containing 13 to 16 predicted transmembrane (TM) domains (Supplemental Fig. S2, A and B), similar to the experimentally confirmed 14 TM domains in *Sc*DOA10 (Fig. 1A) (Kreft et al. 2006).

To determine whether *At*DOA10A and *At*DOA10B represent functional homologs of *Sc*DOA10, we assessed their capacity to complement the yeast *Scdoa10Δ* mutant. We cloned both proteins as C-terminal GFP fusions driven by the *GPD* promoter, transformed them into *Scdoa10Δ*, and confirmed expression using RT-PCR (Fig. 1C). In growth assays, *Scdoa10Δ* displayed relative insensitivity to hygromycin, which was reverted in mutants expressing *At*DOA10A-GFP (to a greater extent than wild-type [WT] yeast, possibly due to overexpression of the transgene), but unaffected in lines expressing *At*DOA10B-GFP (Fig. 1D). Thus, *At*DOA10A, but not the C-terminally truncated *At*DOA10B (Fig. 1A), is able to compensate for the loss of endogenous *Sc*DOA10 activity in yeast, indicating at least partial conservation of function for this putative ortholog.

To understand the nature of the *Arabidopsis* DOA10 gene duplication, we constructed a phylogenetic tree of DOA10-like protein sequences identified via BLASTP from a range of diploid flowering plant genomes, including diverse monocots, dicots, and several members of the Brassicaceae family (Fig. 1E). We found that many, though not all, plant species had two DOA10-like proteins. While most DOA10-like sequences clustered into defined branches that split the monocots and dicots, *At*DOA10B was part of a separate Brassicaceae-specific clade, grouping with similar DOA10B-like sequences from lyre-leaved rock cress (*Arabidopsis lyrata*), field mustard (*Brassica rapa*), and pink shepherd's purse (*Capsella rubella*). In contrast, where two putative orthologs were identified in other species, both sequences occurred together in species-specific pairs within the main *At*DOA10A-like clade—e.g. pineapple (*Ananas comosus*), poplar (*Populus trichocarpa*), and soybean (*Glycine max*). This suggests that the truncated DOA10B-like variant emerged in the Brassicaceae lineage.

### *At*DOA10A and *At*DOA10B are broadly expressed and localize to the endoplasmic reticulum

**Figure 2.**
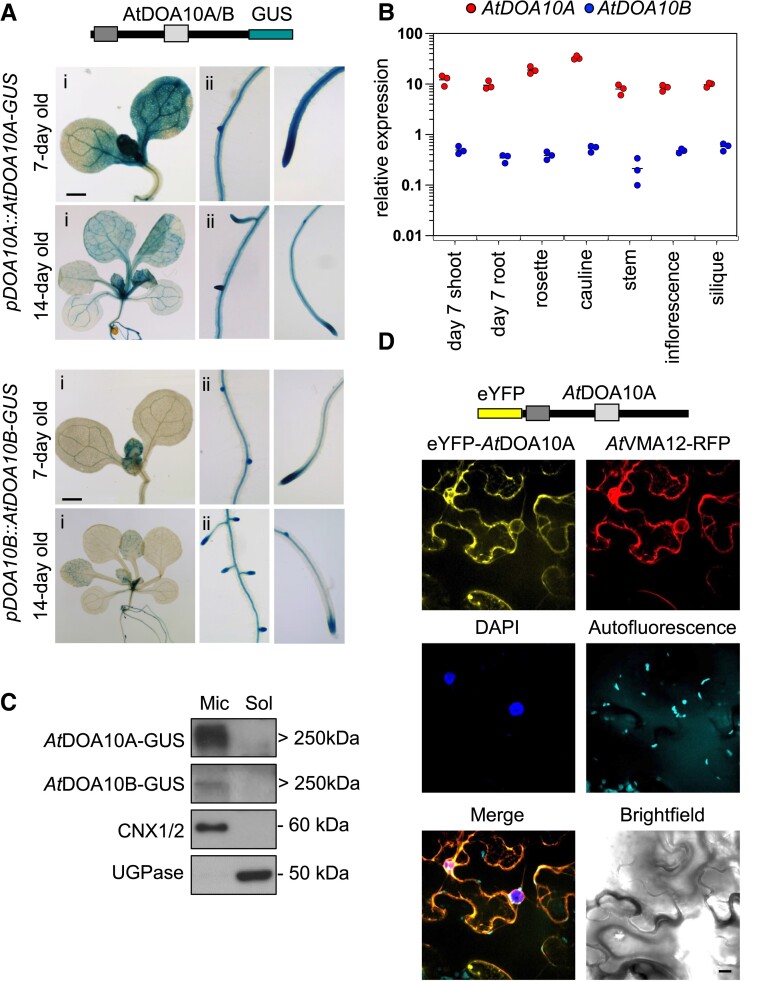
*At*DOA10A and *At*DOA10B are broadly expressed in seedlings and localize to the endoplasmic reticulum. **A)** Histochemical staining of 7- and 14-day-old *Arabidopsis* seedlings expressing *pDOA10A/B::AtDOA10A/B-GUS*. Scale bar for all images: 1 mm. **B)** RT-qPCR of endogenous *AtDOA10A* and *B* mRNA in different seedlings and adult tissues (note the *y* axis Log_10_ scale). Relative expression levels were calculated through normalization to *AtACT7* and are the average of three biological repeats. Horizontal line shows mean. **C)** anti-GUS immunoblot of microsomal and soluble protein extracts from seedlings expressing *pDOA10A/B::AtDOA10A/B-GUS* (expected sizes: *At*DOA10A-GUS, ∼195 kDa; *At*DOA10B-GUS, ∼170 kDa—although both are detected at around 250 kDa). Anti-CNX1/2 (microsomal) and anti-UGPase (soluble) control blots confirming efficacy of the fractionation are shown. **D)** Confocal images of *N. benthamiana* leaf pavement cells transiently co-expressing eYFP-*At*DOA10A and the ER marker protein *At*VMA12-RFP showing co-localization of YFP and RFP signals. Nuclei are stained with DAPI, and chloroplast auto-fluorescence is also shown. Scale bar: 10 *µ*m.

We developed transgenic lines expressing C-terminal GUS fusions of *At*DOA10s driven by 2 kb of the endogenous promoter (*pAtDOA10A/B::AtDOA10A/B-GUS*). Histochemical staining revealed that both proteins are broadly detectable in 7- and 14-day-old seedlings, particularly in roots, which showed enrichment in the primary root meristem, lateral root primordia, and vasculature (Fig. 2A). A complementary reverse transcription quantitative PCR (RT-qPCR) analysis of relative *AtDOA10A/B* mRNA abundance also identified transcripts for both proteins across a range of seedling and adult tissues, suggesting broad roles for *At*DOA10s in diverse cell types (Fig. 2B). Corroborating the reduced levels of *At*DOA10B-GUS staining relative to AtDOA10A-GUS, *AtDOA10B* mRNA abundance was much lower than *AtDOA10A* (approx. 20-fold) across all tissue types.

To determine *At*DOA10 subcellular localization, we isolated total protein from the *pAtDOA10A/B::AtDOA10A/B-GUS* transgenics and prepared soluble and microsomal fractions. Anti-GUS immunoblotting revealed exclusive enrichment of *At*DOA10A/B-GUS in the microsomal fractions, alongside the known ER marker calnexin (CNX) 1/2 (Fig. 2C). We also examined the subcellular localization of an eYFP-*At*DOA10A transgene in transiently transformed *Nicotiana benthamiana* leaf epidermal cells. Here, eYFP-*At*DOA10A co-localized with the ER marker *At*VMA12-RFP (Fig. 2D). Thus, *At*DOA10s are ER-localized, like yeast *Sc*DOA10 and human MARCH6/TEB4.

### Generation and phenotypic assessment of *AtDOA10A* and *B* mutants

**Figure 3.**
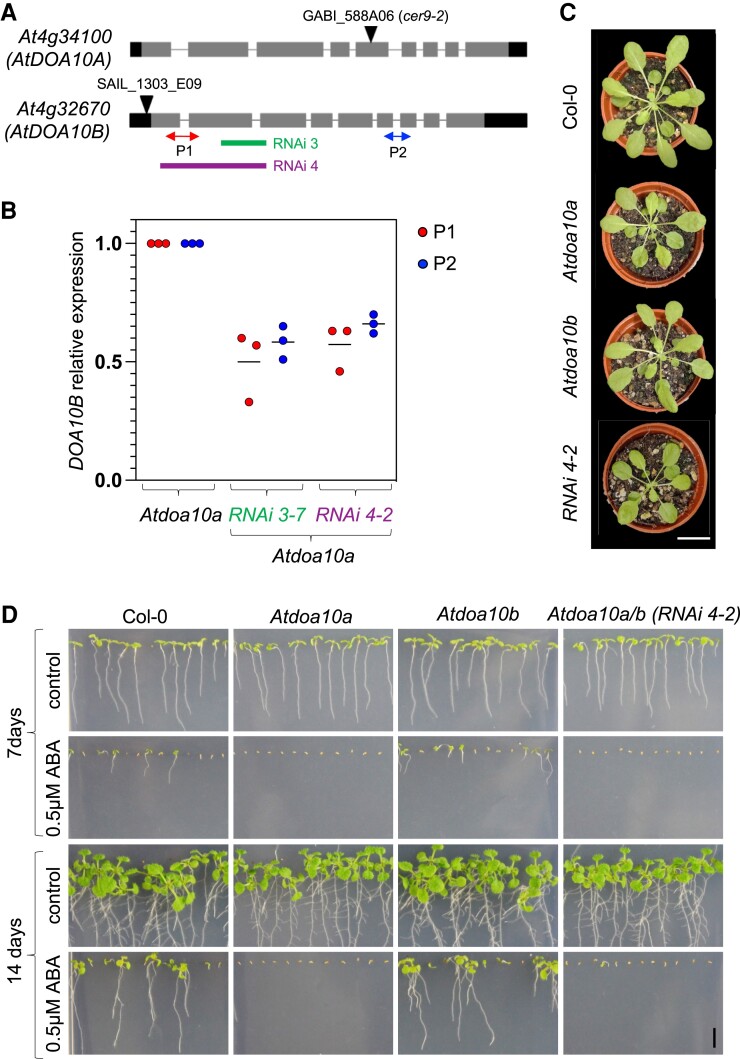
Generation and phenotypic assessment of *At*DOA10A and B mutants. **A)** Schematic of *AtDOA10A* and *B* genes, showing 5′/3′ UTR (black) and exons (grey), T-DNA IDs and insertion sites, position of RNAi construct sequences, and RT-qPCR primers used in (B). **B)** RT-qPCR of endogenous *AtDOA10B* in the *Atdoa10A* control line and homozygous RNAi lines *3-7* and *4-2*, using primer pairs (P1 and P2) upstream and downstream of the RNAi target sequence. Expression levels were normalized to *AtACT7*, and expression in the RNAi lines is shown relative to the endogenous levels of *DOA10B* in the untransformed *Atdoa10A* mutant. Data are the average of three biological repeats. Horizontal line shows the mean. **C)** Rosettes of 6-week-old WT, *Atdoa10a*, *Atdoa10b*, and *Atdoa10a/b RNAi 4-2* lines grown under short days. Images were digitally extracted for comparison. Scale bar: 2 cm. **D)** 7- and 14-day-old seedlings grown on control or 0.5 *µ*M ABA-supplemented ½ MS plates. Scale bar: 5 mm.

We obtained homozygous *Atdoa10a* and *Atdoa10b* T-DNA insertional mutants and confirmed the knockouts by RT-PCR (Fig. 3A and Supplemental Fig. S3A). Although only *At*DOA10A was able to complement *Scdoa10Δ*, we postulated that both AtDOA10s could have overlapping or redundant functions in *Arabidopsis*. As such, we attempted to make a double mutant, but the proximity of the two genes (separated by 0.57 Mb) meant that a crossover event was likely to be very rare; in accordance with this, we were unable to identify any double mutants and instead took an RNAi approach. We designed two different RNAi constructs that targeted the first and second exons of *AtDOA10B* (Fig. 3A) and transformed these into *Atdoa10a*. RT-qPCR analysis of the derived progeny led to the identification of two independent lines with reduced *AtDOA10B* mRNA (∼50% Col-0 levels): *Atdoa10a/b RNAi 3-7* and *RNAi 4-2* (Fig. 3B). Despite screening several lines, we did not identify any with stronger depletion.

No major phenotypic differences between lines were observed when grown under standard conditions, though mutant rosettes were slightly smaller than WT rosettes, in accordance with previous observations for *Atdoa10a* (Fig. 3C) (Huber et al. 2022). *At*DOA10A was previously linked to ABA signaling and the control of cuticle development, with *Atdoa10a* mutants displaying seed ABA hypersensitivity and drought tolerance phenotypes (Lü et al. 2012; Zhao et al. 2014). We also observed ABA hypersensitivity of *Atdoa10a* seeds (Fig. 3D) and showed that WT sensitivity was restored in *Atdoa10a* lines complemented with *pDOA10A::AtDOA10A-YFP* (Supplemental Fig. S3B). In contrast, the *Atdoa10b* single mutant had no obvious phenotype, and ABA sensitivity of *Atdoa10a/b RNAi 4-2* resembled that of the *Atdoa10a* single mutant, suggesting that *At*DOA10A and *At*DOA10B do not have additive or redundant roles in ABA-related responses.

### RNA-sequencing and Nt-acetylome profiling indicate that *At*DOA10s do not regulate global turnover of Nt-acetylated proteins

**Figure 4.**
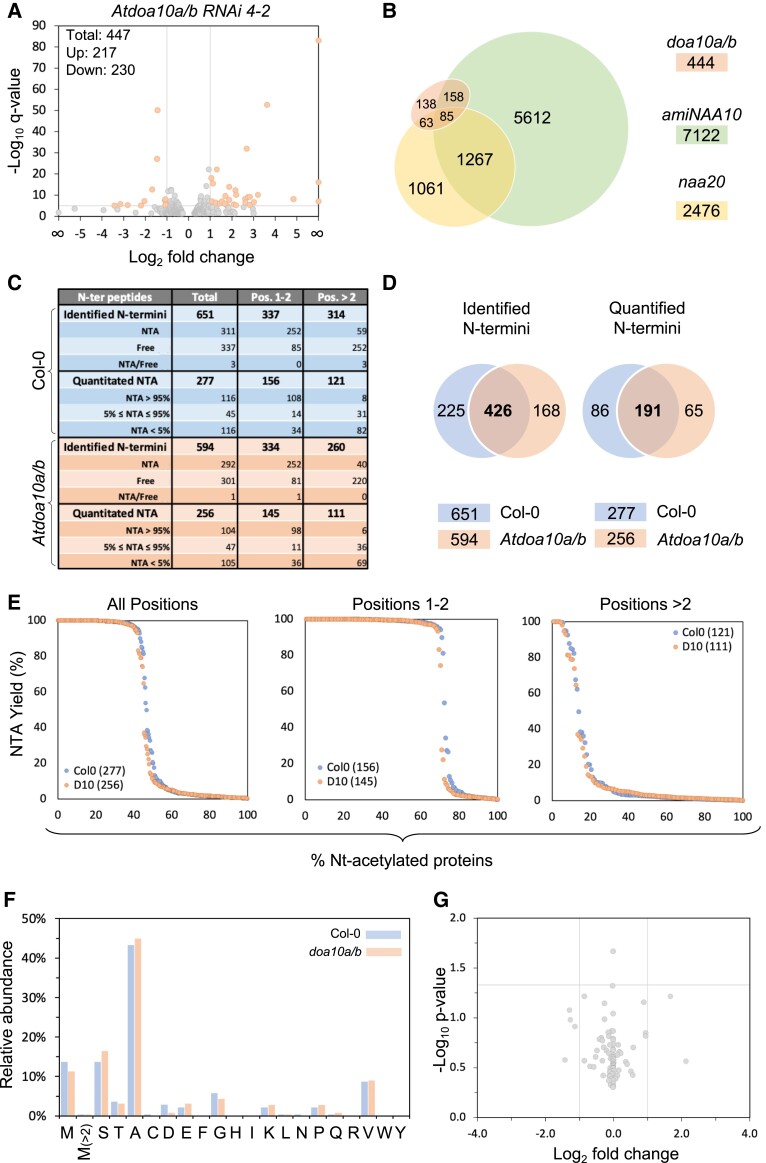
RNA-seq and Nt-acetylome profiling indicate that AtDOA10s do not regulate global turnover of Nt-acetylated proteins. **A)** Volcano plot of up and down DEGs in 10-day-old seedlings of *Atdoa10a/b RNAi 4*-*2* vs Col-0. Orange data points represent mRNAs that are >2-fold up or down (log_10_(q) > 5). **B)** Venn diagram showing overlap in total DEGs (excluding non-annotated mRNAs) relative to Col-0 between 10-day-old seedlings of *Atdoa10a/b RNAi 4*-*2*, *amiNAA10*, and *naa20.***C)** Summary table of N-terminal profiling. **D)** Venn diagrams showing numbers and overlap of (i) identified N-termini and (ii) quantified N-termini in *Atdoa10a/b RNAi 4-2* vs Col-0. **E)** Global NTA variation comparison in *Adoa10a/b RNAi 4-2* and Col-0. For each sample, the peptides were sorted in decreasing order of %NTA (quantitated only). Each Nt-peptide was assigned a number corresponding to its relative position. These protein numbers are plotted with matching NTA yield (%), either for all Nt-acetylation positions, protein Nt-positions (pos. 1 or 2), or downstream Nt-acetylation (pos. >2). **F)** Relative abundance of acetylated Nt-residues (shown as %) in *Atdoa10a/b RNAi 4-2* and Col-0. **G)** Relative comparison of NTA levels between *Atdoa10a/b RNAi 4-2* and Col-0, for peptides quantified in both data sets, showed no significant (*P* < 0.05) differences.

We carried out an RNA-seq analysis of 10-day-old *Atdoa10a/b RNAi 4-2* seedlings grown under long-day conditions, which identified 447 differentially expressed genes (DEGs; 217 up, 230 down) relative to Col-0 (False Discovery Rate [FDR] adjusted *P*-value [q] of <0.05) (Fig. 4A and Supplemental Data Set S1). Among these, 89 had a fold change of 2 or more. This modest number of DEGs likely reflects the fact that plants were grown under ambient, non-stressed conditions. Nonetheless, Gene Ontology (GO) analysis uncovered several enriched GO terms that are consistent with potential roles for *At*DOA10s in protein quality control or ERAD, including cellular component term *perinuclear region of the cytoplasm* (18.1-fold enrichment, 2.05E-07 FDR), biological process terms *protein folding* (4.63-fold enrichment, 6.31E-03 FDR) and *protein refolding* (10.18-fold enrichment, 1.50E-02 FDR), and molecular function term *unfolded protein binding* (5.14-fold enrichment, 1.61E-02 FDR). We also carried out RNA-seq analysis on 10-day-old NAT-depleted plants: *amiNAA10* (i.e. *NATA*) knockdown and *Atnaa20* (i.e. *NATB*) mutant seedlings. We hypothesized that there might be some overlap in the transcriptome of *Atdoa10a/b* and *nat* mutants, due to shared ectopic stabilization of Ac/N-degron protein targets. As expected, given NATA's role in acetylating 40% of cytosolic proteins, *amiNAA10* seedlings had the greatest number of DEGs (7,139), with *Atnaa20* having fewer (2,486). Interestingly, nearly 70% of the *Atdoa10a/b* RNAi-annotated DEGs (307/444) were also differentially expressed in either NAT mutant transcriptome (Fig. 4B), with 75% of *Atdoa10a/b*-*amiNAA10* and 78% of *Atdoa10a/b*-*naa20* shared DEGs occurring in the same direction (i.e. up in both or down in both; Supplemental Data Set S1).

If *At*DOA10s function as general Ac/N-recognins of the Ac/N-degron pathway, an accumulation of Nt-acetylated proteins would be expected in plants lacking *At*DOA10 function compared to Col-0. This could manifest as either an increase in the total levels of Nt-acetylated proteins or increased ratio of acetylated to non-acetylated variants of a particular protein(s). We performed quantitative Nt-acetylome profiling on total protein extracts from both Col-0 and *doa10a/b RNAi 4-2* seedlings using the “stable isotope labeling protein N-terminal acetylation quantification (SILProNAQ)” method (Bienvenut et al. 2017a), with data processed using the EnCOUNTer tool (Bienvenut et al. 2017b) (Supplemental Data Set S2). A total of 651 and 594 N-termini were identified in Col-0 and *Atdoa10a/b RNAi 4-2*, respectively, with 426 common to both lines (Fig. 4, C and D). In both genotypes, approximately half of these N-termini were Nt-acetylated (48% in Col-0 and 49% in *Atdoa10a/b*). We were able to quantify a total of 342 unique N-termini (277 in Col-0 and 256 in *Atdoa10a/b RNAi 4-2*). In Col-0 58% (161/277) were either fully or partially acetylated, which was similar to 59% observed for *Atdoa10a/b RNAi 4-2* (151/256). Global NTA variation comparisons based on either N-terminal position (Fig. 4E) or cellular sub-compartment (Supplemental Fig. S4) showed no differences in overall NTA level in *Atdoa10a/b RNAi* 4-2 vs Col-0, and a similar distribution in NTA-frequency was observed for all natural amino acids at the N-terminal position (Fig. 4F). Moreover, relative quantification of Nt-acetylated peptides shared between *Atdoa10a/b RNAi 4-2* and WT identified no proteins with significantly (i.e. FDR < 5%) increased or decreased NTA (Fig. 4G). Collectively, these Nt-profiling findings suggest that strong depletion of *At*DOA10 levels does not affect the overall turnover of Nt-acetylated proteins.

### Proteolytic turnover of *At*SQE1 requires *At*DOA10A and *At*NAA20 in heterologous yeast degradation assays

While the *Atdoa10a/b RNAi 4-2* double mutant had no clear differences in global NTA, *At*DOA10s could still play a role in targeting specific Nt-acetylated protein substrates for degradation. We took a targeted approach to identify a potential physiological substrate to use for investigating protein turnover in finer molecular detail. Mutations in *At*DOA10A were previously shown to suppress phenotypic defects in the *dry2* mutant, which lacks SQE1 activity. Here, defects arise due to a build-up of toxic intermediates, and a secondary mutation in *At*DOA10A alleviates this by downregulating HMGR enzyme activity, which functions several steps ahead of squalene synthesis (Doblas et al. 2013). Moreover, in yeast and mammals, SQE homologs (Ergosterol Biosynthesis 1 [ERG1] in yeast and squalene monooxygenase [SM] in mammals) localize to the ER membrane and are direct proteolytic targets of *Sc*DOA10 and *Hs*MARCH6/TEB4, respectively (Foresti et al. 2013; Zelcer et al. 2014; Sharpe et al. 2020). Similarly, *Arabidopsis* SQE1 is predicted to have transmembrane regions and localize to the ER, although it does not contain a predicted N-terminal secretory signal peptide (Supplemental Fig. S2, C and D). Given the evolutionary conservation of DOA10-like E3 ligases and their roles in modulating sterol biosynthetic pathways, we postulated that *Arabidopsis* SQE1 turnover might also be regulated by *At*DOA10s.

**Figure 5.**
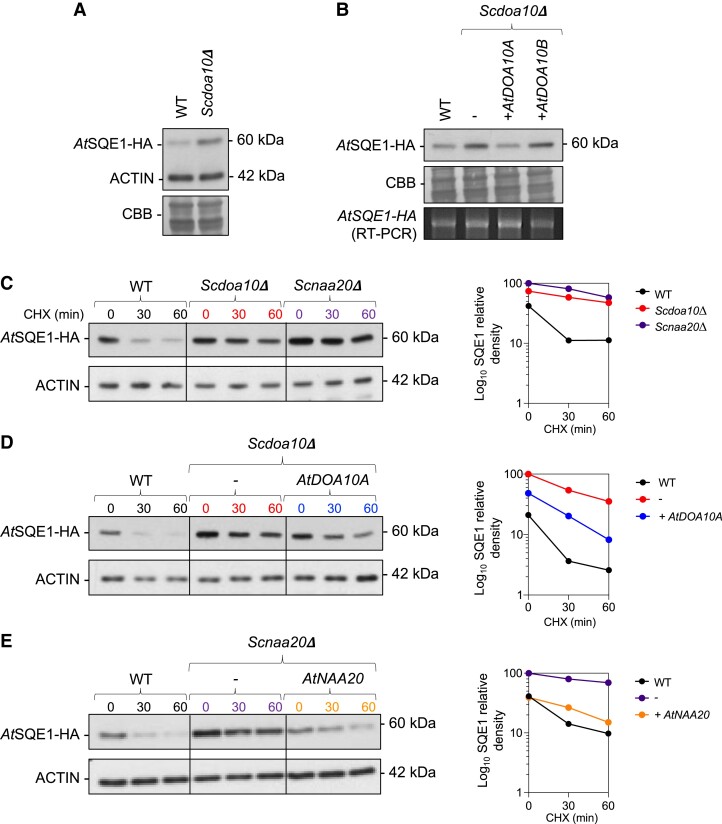
Proteolytic turnover of *At*SQE by *At*DOA10A and *At*NAA20 in heterologous yeast degradation assays. **A)** Anti-HA immunoblot showing steady-state levels of AtSQE1-HA in WT vs *Scdoa10Δ* yeast cells. Anti-ACTIN bands are shown on the same blot. CBB, Coomassie Brilliant Blue loading control. **B)** Steady-state protein (immunoblot) and mRNA (RT-PCR) levels of AtSQE1-HA expressed in WT vs *Scdoa10Δ* ± co-expression with *At*DOA10A or *At*DOA10B. **C)** Cycloheximide (CHX) chase of *At*SQE1-HA in WT, *Scdoa10Δ*, and *Scnaa20Δ* yeast cells (immunoblot and quantified relative density). **D)** Cycloheximide chase showing that co-expression of *At*DOA10A destabilizes *At*SQE1-HA in *Scdoa10Δ* yeast cells (immunoblot and quantified relative density). **E)** Cycloheximide chase showing that co-expression of *At*NAA20 destabilizes *At*SQE1-HA in *Scnaa20Δ* yeast cells (immunoblot and quantified relative density).

We used WT and mutant yeast strains as a heterologous system for expressing *At*SQE1 and monitoring its stability by immunoblotting. Steady-state levels of *At*SQE1-HA were higher in *Scdoa10Δ* than WT yeast, and this could be reverted when *At*SQE1-HA was co-expressed with *At*DOA10A-YFP, but not *At*DOA10B-YFP (Fig. 5, A and B). Next, we monitored *At*SQE1-HA turnover rates using cycloheximide (CHX) chase assays, which revealed rapid degradation of *At*SQE1-HA in WT yeast cells, but relative stabilization in *Scdoa10Δ* (Fig. 5C). Moreover, the enhanced stability was partially reverted when *At*DOA10A-YFP was co-transformed into *Scdoa10Δ* (Fig. 5D).

*At*SQE1 initiates with the residues Met-Glu- (ME-) and should retain its initiator Met during translation and be targeted by ribosome-associated NATB (comprising NAA20 catalytic and NAA25 auxiliary subunits) (Aksnes et al. 2019). It was previously shown that 99% of ME-initiating proteins undergo NTA in humans (Aksnes et al. 2016), with similarly high numbers in yeast and *Arabidopsis* (Supplemental Fig. S5 and Supplemental Data Set S3). It was also shown that human *HsNAA20* can complement *Arabidopsis Atnaa20* mutants but that yeast *ScNAA20* cannot (Huber et al. 2020). Whether *At*NAA20 can compensate for loss of *Sc*NAA20 function is unknown. To investigate whether *At*SQE1-HA turnover is mediated by Nt-acetylation and the Ac/N-degron pathway, we also monitored *At*SQE1-HA stability in the yeast *Scnaa20Δ* mutant (Fig. 5, C and E). Similar to *Scdoa10Δ*, and potentially consistent with the Ac/N-degron pathway, we saw strong enhancement of *At*SQE1-HA stability relative to WT, which was almost completely reverted when *At*NAA20 was co-expressed. This indicates that NATB activity is required for *At*SQE1 turnover and reveals that *Arabidopsis At*NAA20 can functionally replace *Sc*NAA20.

### Impact of N-terminal mutagenesis on *At*SQE1 stability suggests indirect effects of Nt-acetyltransferases on protein turnover in yeast

**Figure 6.**
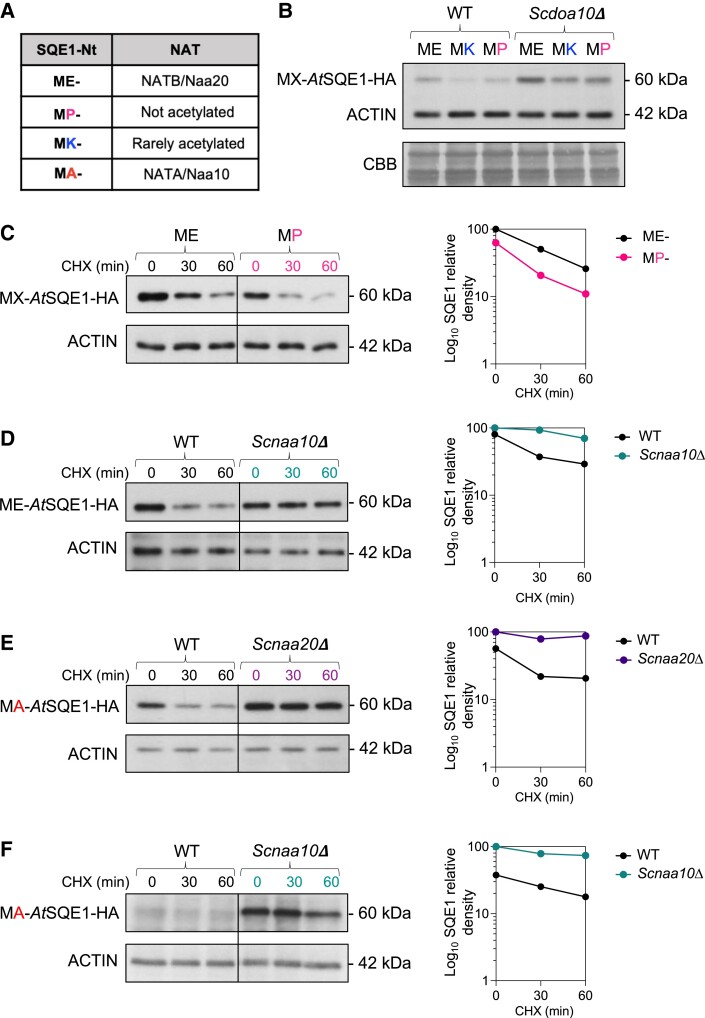
Impact of N-terminal mutagenesis on AtSQE1 stability suggests indirect effects of Nt-acetyltransferases on protein turnover in yeast. **A)** Summary of N-terminal (Nt) mutants and predicted respective NAT activities. **B)** Steady-state protein levels of *At*SQE1-HA Nt-variants in WT and *Scdoa10Δ* yeast cells. **C)** Cycloheximide (CHX) chase of WT ME- and mutant MP-*At*SQE1-HA in WT yeast cells (immunoblot and quantified relative density). **D)** Cycloheximide (CHX) chase of WT ME-*At*SQE1-HA in WT and *Scnaa10Δ* yeast cells (immunoblot and quantified relative density). **E)** Cycloheximide (CHX) chase of mutant MA-*At*SQE1-HA in WT and *Scnaa20Δ* yeast cells (immunoblot and quantified relative density). **F)** Cycloheximide (CHX) chase of mutant MA-*At*SQE1-HA in WT and *Scnaa10Δ* yeast cells (immunoblot and quantified relative density).

To further investigate the connection between NTA and *At*SQE1 degradation, we developed a series of Nt-mutagenized variants of *At*SQE1 that are predicted to be blocked for NTA or which are targeted by different cognate NATs (Fig. 6A, Supplemental Fig. S5, and Supplemental Data Set S3): (1) MP-SQE1, which should undergo Met-excision by methionine aminopeptidases (MetAPs) but no further Nt-acetylation, as Nt-Proline residues are not acetylated (Goetze et al. 2009); (2) MK-SQE1, which should be rarely acetylated (Arnesen et al. 2009); and (3) MA-SQE1, which would instead be Nt-acetylated by NATA/NAA10 following initiator Met removal by MetAP. We hypothesized that MP- and MK-SQE1-HA would be stable in WT yeast if turnover is dependent on NTA but instead found that their steady-state levels were in fact reduced relative to WT ME-*At*SQE1-HA (Fig. 6B), perhaps due to codon differences impacting translation (Kozak 1997). Moreover, when expressed in *Scdoa10Δ*, relative abundance increased for all proteins, but the ratios between them were maintained, suggesting that *Sc*DOA10 targets all three Nt-variants and that their direct NTA is not critical for degradation.

In accordance with these steady-state analyses (Fig. 6B), we found that mutant MP-*At*SQE1-HA rapidly degraded following CHX treatment, similar to WT ME-*At*SQE1-HA (Fig. 6C). We also observed stabilization of WT ME-*At*SQE1-HA in *Scnaa10Δ* mutant cells (Fig. 6D), which lack a functional NATA enzymatic complex; this was unexpected, since ME- is not a target sequence for NATA activity. Finally, we examined the stability of mutant MA-*At*SQE1-HA, where the N-terminus is remodeled from a NATB to a NATA target. Like all other variants, MA-*At*SQE1-HA was unstable in WT yeast but was surprisingly still stable in the non-cognate Sc*naa20Δ* mutant in addition to *Scdoa10Δ* (Fig. 6, E and F). Collectively, these assays indicate that degradation of *At*SQE1 via DOA10 in yeast is indirectly influenced by NATA and NATB activity.

### Nt-acetylation-independent turnover of *At*SQE1 by *At*DOA10 in *Arabidopsis*

**Figure 7.**
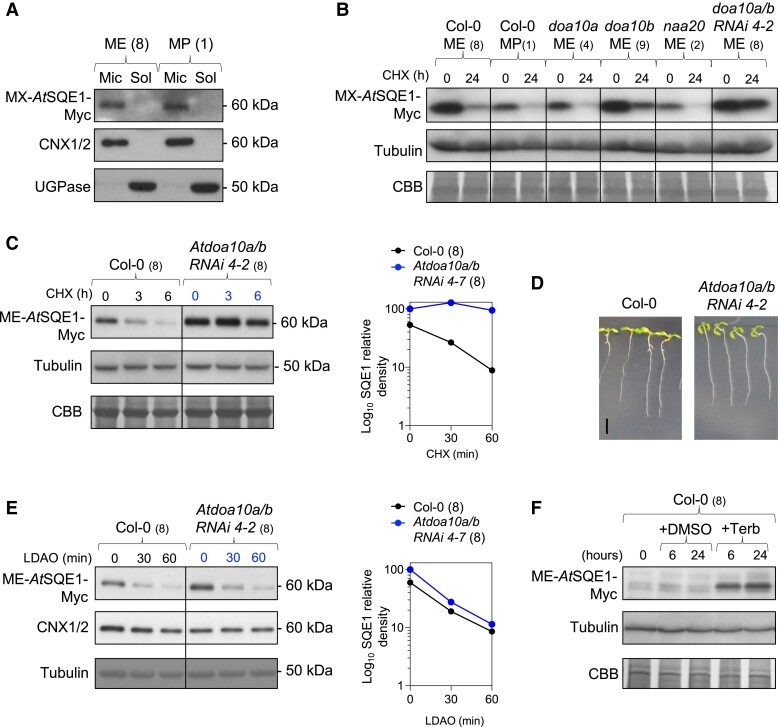
Nt-acetylation-independent turnover of *At*SQE1 by *At*DOA10 in *Arabidopsis*. **A)** Microsomal and soluble protein extracts from seedlings expressing WT ME- and mutant MP-*At*SQE1-Myc. Anti-CNX1/2 (microsomal) and anti-UGPase (soluble) control blots confirming efficacy of the fractionation are shown. **B)** Cycloheximide (CHX) chase of WT ME- and mutant MP-*At*SQE1-Myc variants in WT Col-0 and different mutant backgrounds. Independent transgenics have different starting expression levels (see Supplemental Fig. S6A), and so comparisons of protein levels can only be directly made between time points within lines. **C)** Cycloheximide (CHX) chase of WT ME-*At*SQE1-HA in Col-0 and *Atdoa10a/b RNAi 4-2* seedlings (immunoblot and quantified relative density). **D)** 7-day-old seedlings on ½ MS showing epinasty in *Atdoa10a/b RNAi 4-2*. Scale bar: 5 mm. **E)** LDAO chase of WT ME-*At*SQE1-Myc in Col-0 and *Atdoa10a/b RNAi 4-2* seedlings (immunoblot and quantified relative density). **F)** Terbinafine chase of WT ME-*At*SQE1-Myc in Col-0.

To test *At*SQE1 stability *in planta*, we generated a range of stable transgenic *Arabidopsis* lines expressing Nt-variants of *At*SQE1-Myc driven by the constitutive 35S CaMV promoter. WT ME-*At*SQE1-Myc was expressed in Col-0, *Atdoa10a*, *Atdoa10b*, *Atdoa10a/b RNAi 4-2*, and *Atnaa20*, while mutant non-acetylatable MP-AtSQE1-Myc was expressed in Col-0 only. For each construct we identified two independent transgenics and confirmed their expression by RT-qPCR (Supplemental Fig. S6A). Both the ME- and MP- Nt-variants of *At*SQE1-Myc localized to the ER in Col-0 (Fig. 7A). Thus, *At*SQE1 resides in the same cellular compartment as *At*DOA10s (like in yeast and mammals), and the introduced E2P N-terminal mutation does not disrupt this subcellular targeting. CHX chase assays revealed that WT ME-*At*SQE1-Myc was unstable after 24 h of translational shutdown with CHX in all genetic backgrounds tested, except for the *Atdoa10a/b RNAi 4-2* double mutant (Fig. 7B). This was consistent across two independent transgenic lines (Fig. 7B and Supplemental Fig. S6B). A shorter CHX experiment corroborated this finding, showing that WT ME-*At*SQE1-Myc is turned over in WT within 6 h, but remains stable in the *Atdoa10a/b RNAi 4-2*, or if co-incubated with proteasome inhibitor bortezomib (Fig. 7C and Supplemental Fig. S6C). Mutant MP-*At*SQE1-Myc was also unstable in Col-0 (Fig. 7B). These data reveal that proteasomal degradation of *At*SQE1 requires both *At*DOA10 proteins *in planta* and that this turnover is neither dependent on indirect NAT activity (in contrast to yeast; Figs. 5 and 6) nor direct NTA of *At*SQE1 by NATB.

Potentially consistent with the fact that *At*SQE1 accumulates in *Atdoa10a/b RNAi 4-2*, we observed extreme epinasty in seedlings of this mutant relative to Col-0, a phenotype that has previously been shown to coincide with altered sterol biosynthesis (Fig. 7D) (Carland et al. 2010). Precise control of sterol synthesis pathway enzymes is required to regulate and maintain appropriate levels of phytosterols and their intermediates in *Arabidopsis* (De Vriese et al. 2021). In human and yeast systems, SQE expression and stability are influenced by the accumulation or consumption of different sterols and pathway intermediates, but how such feedback works in *Arabidopsis* is currently unknown (Leber et al. 2001; Gill et al. 2011; Foresti et al. 2013; Scott et al. 2020). We investigated whether chemical inhibition of *Arabidopsis* sterol synthesis impacts *At*SQE1 stability via *At*DOA10s through treating seedlings with the chemical lauryldimethylamine oxide (LDAO), a dual inhibitor of both the cycloartenol synthase (CAS) enzymes, which cyclize 2,3-oxidosqualene (the direct product of *At*SQE1) to produce cycloartenol, and cycloeucalenol cycloisomerase (CPI) enzymes, which act further downstream (Darnet et al. 2020). We observed extremely rapid turnover of ME-*At*SQE1-Myc protein in response to LDAO treatment, occurring within just 30 min (Fig. 7E). This was specific to ME-*At*SQE1-Myc and not a consequence of general protein turnover nor ER disruption due to LDAO's detergent properties, since ER-localized CNX1/2 proteins were unaffected. Interestingly however, LDAO was still able to induce rapid turnover of ME-*At*SQE1-Myc in *Atdoa10a/b RNAi 4-2* (Fig. 7E). We also directly inhibited AtSQE1 using terbinafine, a non-competitive inhibitor of squalene epoxidase enzymes (Nowosielski et al. 2011); this led to strong accumulation of *At*SQE1-Myc (Fig. 7F), likely caused by positive feedback through reduced degradation given that expression was driven by the constitutive 35S CaMV promoter.

## Discussion

In yeast and humans, DOA10 E3 ligases function as Ac/N-recognins that target functionally diverse proteins for degradation via recognition of their acetylated N-termini (Hwang et al. 2010; Shemorry et al. 2013; Park et al. 2015). In plants, the Ac/N-degron pathway and its associated Ac/N-recognins are yet to be characterized, although it was previously shown that NTA of rice HYPK and a specific Nt-isoform of *Arabidopsis* SNC1 triggers their degradation (Xu et al. 2015; Gong et al. 2022). While HYPK and SNC1 are potential plant Ac/N-degron pathway targets, the *Arabidopsis* SIB1 protein was recently shown to be stabilized by NATB-mediated NTA (Li et al. 2020). Moreover, large-scale studies in yeast, humans, and plants have established broader roles for NTA in proteome stabilization (Kats et al. 2018; Friedrich et al. 2021; Mueller et al. 2021; Gibbs et al. 2022; Linster et al. 2022). Therefore, the relationship between protein NTA and degradation is complex and is likely to vary depending on protein identity and cellular context. Here, we investigated *Arabidopsis At*DOA10-like E3 ligases, focusing specifically on their potential function as Ac/N-recognins. Nt-acetylome profiling revealed no apparent differences in the accumulation of Nt-acetylated proteins in *Atdoa10a/b RNAi* vs Col-0, suggesting that depletion of *At*DOA10 proteins does not influence the bulk turnover of Nt-acetylated proteins. However, the proteins identified and quantified in our comparative Nt-acetylome analysis represent only a small proportion of total potential Ac/N-degron substrates, which means we cannot rule out that *At*DOA10s may have specific roles in targeting a more constrained set of Nt-acetylated proteins. Alternatively, other E3 ligases, for example, putative orthologs of the NOT4 Ac/N-recognins (Shemorry et al. 2013; Gibbs et al. 2016), may function as primary Ac/N-recognins in plants.

We identified the ER-resident *At*SQE1 protein, a rate-limiting enzyme in the sterol biosynthesis pathway (Rasbery et al. 2007), as a target of *At*DOA10s. Turnover of *At*SQE1 requires functional *At*DOA10s and was not linked to acetylation of its N-terminus *in planta*, since its stability was unaltered when its putative cognate NAT, NATB, was deleted (Fig. 7B). Surprisingly however, we found that *At*SQE1 stability was indirectly influenced by NAT activity when heterologously expressed in yeast. Partial or complete NATA or NATB inactivation led to enhanced stabilization regardless of the Nt-variant being assayed (Figs. 5 and 6), which may be due to indirect effects on other proteins linked to translational or proteolytic machineries; the ERAD protein *Sc*Der1, for example, requires NTA by NATB for stability (Zattas et al. 2013). Our cross-kingdom analysis of *At*SQE1 stability has therefore uncovered differences in the ways in which NTA can influence proteostasis in yeast and plants, suggesting that caution should be applied when investigating the connection between NTA and protein turnover and particularly when extrapolating between organisms.

In contrast to yeast and humans, the *Arabidopsis* genome encodes for two DOA10-like E3 ligases. *At*DOA10A, but not *At*DOA10B, was able to complement *Scdoa10Δ* yeast, with respect to both hygromycin sensitivity and turnover of heterologously expressed *At*SQE1 (Figs. 1 and 5). While both *At*DOA10s are smaller than *Sc*DOA10, *At*DOA10B is particularly truncated (Fig. 1A) and appears to be restricted to the Brassicaceae clade (Fig. 1E), which could explain its inability to complement *Scdoa10Δ*. This may be the result of incompatibility between *At*DOA10B and components of the endogenous yeast ubiquitination machinery, since in *Arabidopsis At*SQE1 degradation was only inhibited in the *Atdoa10a/b RNAi* double mutant. Despite this example of functional redundancy, other evidence points to additional paralog-specific activities. For instance, *Atdoa10a* single mutants display a range of phenotypes that do not manifest in *Atdoa10b* and which are not amplified in *Atdoa10a/b RNAi* lines, including altered cuticular wax composition and strong ABA hypersensitivity (Fig. 3D) (Lü et al. 2012; Zhao et al. 2014).

DOA10s in yeast and humans are major E3 ligases of the ERAD system (Ravid et al. 2006), while roles for AtDOA10s in the *Arabidopsis* ERAD system are still unclear (Liu et al. 2011; Li et al. 2017; Huber et al. 2022). This may be due to functional redundancy, a concept supported by our observations that turnover of ER-resident AtSQE1 is dependent on both AtDOA10s. Interestingly though, AtDOA10B was shown to physically associate with the ERAD-associated ubiquitin conjugase 32 (UBC32) (Cui et al. 2012) and be transcriptionally induced by L-azetidine-2-carboxylic acid (AZC), a proline analogue that causes protein misfolding (Kim et al. 2017). We also observed significant upregulation of AtDOA10B, but not AtDOA10A, in response to the ERAD elicitor tunicamycin (Supplemental Fig. S7). This suggests that AtDOA10s have both redundant and distinct roles linked to different cellular processes and that the AtDOA10B paralog may have evolved to take on a more prominent role in stress-associated, rather than constitutive, ERAD in *Arabidopsis*. Further analysis of the transcriptomic, proteomic, and physiological response of *Atdoa10a* and *b* mutants to ER and protein misfolding stresses will shed further light on the roles these proteins play in ERAD and protein homeostasis.

**Figure 8.**
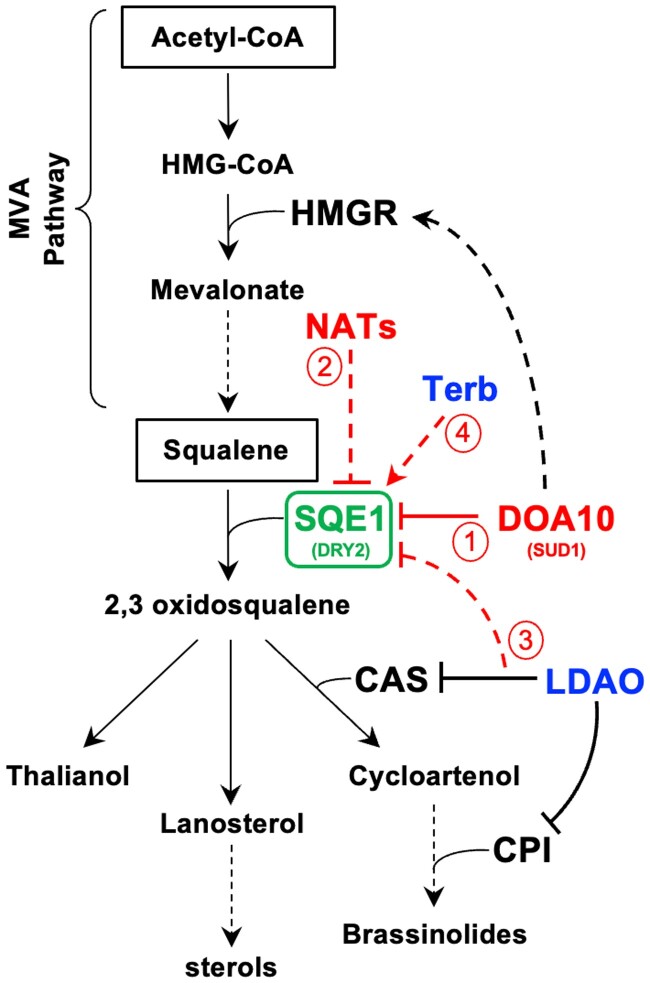
Summary of the mevalonate (MVA) and sterol synthesis pathways in *Arabidopsis*: (1) DOA10 negatively regulates SQE1 stability (this study) and positively regulates HMGR activity (Doblas et al. 2013) in plants, yeast, and humans. Yeast and animal SQEs were previously shown to be targets of DOA10 (Foresti et al. 2013; Zelcer et al. 2014), indicating conservation of this regulatory module across three eukaryotic kingdoms. (2) NATA and B were shown to indirectly contribute to AtSQE1 turnover in yeast (this study), but not in *Arabidopsis*. (3) LDAO, an inhibitor of several downstream enzymatic steps, also negatively regulates AtSQE1 levels via DOA10-independent mechanism(s). (4) Terbinafine, a chemical inhibitor of SQE enzymatic activity (Nowosielski et al. 2011), indirectly promotes accumulation of AtSQE1 (this study), likely through positive feedback. Dashed lines denote indirect effects.

Sterol biosynthesis is sensitive to fluctuations in enzyme activity and substrate availability at each stage. For example, a build-up of cholesterol in animals feeds back to downregulate the pathway and redirect precursor flux, while increases in lanosterol abundance has a similar effect in yeast (Gill et al. 2011; Foresti et al. 2013; Scott et al. 2020). In plants, a build-up of squalene and/or its precursors is toxic but can be counteracted by a reduction of HMGR enzyme activity, which is enhanced when *At*DOA10A is knocked out (Doblas et al. 2013). Our LDAO assays reveal that ectopic build-up of downstream sterol intermediates also triggers feedback mechanisms to downregulate sterol production in plants, in this case promoting *At*SQE1 destabilization, possibly through an alternative E3 ligase or an autophagic mechanism (Fig. 7E). In contrast, direct inhibition of AtSQE1 with terbinafine led to increased accumulation of *At*SQE1 (Fig. 7F). Interestingly, *Sc*DOA10 was previously shown to target multiple enzymes of the sterol biosynthesis pathway in yeast, indicating that it is a master regulator of this biochemical pathway (Scott et al. 2020). The fact that *At*DOA10s influence both HMGR activity and SQE1 stability also points to central functions for DOA10s in coordinating sterol production at several steps in plants (Fig. 8). Whether *At*DOA10s control turnover of other *Arabidopsis* SQEs (Rasbery et al. 2007) remains to be determined, though the Brassicaceae*-*specific *AtSQE5* (Laranjeira et al. 2015) was significantly downregulated in the *Atdoa10a/b RNAi 4-2* transcriptome, potentially suggesting negative feedback. Furthermore, direct interaction and ubiquitination of AtSQE1 by AtDOA10s need to be confirmed biochemically. A more detailed analysis of the connection between *At*DOA10s, *At*SQEs, and other components of the sterol synthesis pathway in *Arabidopsis* will provide further insight into the complexities of sterol homeostasis in plants.

To conclude, our work suggests that DOA10-like E3 ligases, in contrast to their putative yeast and mammalian homologs, do not play a major role in the degradation of Nt-acetylated proteins in *Arabidopsis*, suggesting that the plant Ac/N-degron pathway is less highly conserved across kingdoms than other N-degron pathways and that its E3 ligase component(s) await discovery. Furthermore, we uncover conservation of a DOA10-SQE regulatory module across 1.5 billion years of evolution, which suggests that homeostatic mechanisms controlling sterol biosynthesis have ancient origins.

## Materials and methods

### Arabidopsis growth conditions and transgenic lines

*Arabidopsis* (*Arabidopsis thaliana*) lines were obtained from the Nottingham Arabidopsis Stock Centre (NASC), apart from *amiNAA10* and *naa20* (Linster et al. 2015; Huber et al. 2020). *AtDOA10A/B-GUS/YFP* transgenics were produced by cloning the genomic sequences of *AtDOA10A/B* (At4g34100 and At4g32670), including ∼2 kb of upstream sequence, into the Gateway entry vectors pDONR221/pENTR/D-TOPO (Invitrogen) before ligation into pGWB533/pGWB540 (Nakagawa et al. 2007). *AtDOA10B* RNAi target sequences were cloned into pK7GWIWG2(I) (Karimi et al. 2002). The *AtSQE1* (At1G58440) CDS was cloned into pENTR/D-TOPO (Invitrogen) and subsequently pGWB17 (Nakagawa et al. 2007) to produce *35S::AtSQE1-Myc*; the E2P mutation was introduced via a mismatched forward primer. Expression clones were transformed into *Agrobacterium tumefaciens* (GV3101 pMP90) for *Arabidopsis* transformation via floral dip (Zhang et al. 2006). All cloning and genotyping primers are listed in Supplemental Table S1.

*Arabidopsis* plants were grown on soil (Levington M3 compost, vermiculite, and perlite; 4:2:1 ratio), in long-day (16 h light at 22°C) or short-day (8 h light at 22°C) conditions. For sterile growth, seeds were surface sterilized (10% v/v bleach), plated onto half-strength Murashige & Skoog (½ MS) medium (1% w/v agar, pH 5.7), stratified at 4°C for 48 h, and grown in long days. ABA (Sigma-Aldrich) was added directly into the ½ MS growth medium to the appropriate concentration. LDAO (Cayman Chemical Company) treatments were carried out on 7-day-old seedlings in water supplemented with 100 *µ*g ml^−1^ LDAO. Terbinafine hydrochloride (20 *µ*M) (Sigma-Aldrich) was applied to seedlings in the same manner as cycloheximide (see below). All experiments involving chemical treatment of *Arabidopsis* were conducted at least three times.

### Yeast assays

Yeast (*Saccharomyces cerevisiae*) cells used were homozygous diploid BY4743 cells derived from the S288C strain (Dharmacon yeast KO collection, Horizon Discovery). Yeast were transformed with *AtDOA10A*, *AtDOA10B*, and *AtNAA20* (At1g03150) in the pAG416GPD-ccdB-EGFP vector (Addgene plasmid #14196, Susan Lindquist) and *AtSQE1* in the pAG413GPD-ccdB-HA vector (Addgene plasmid #14238, Susan Lindquist) or the corresponding empty vectors. Transformation was performed by the lithium acetate method: A 1 *µ*L loop of cells was added to 2 *µ*g of expression clone and 100 *μ*L of transformation buffer (33% v/v polyethylene glycol 3350, 0.33 M lithium acetate, 0.66% v/v β-mercaptoethanol). Cells were briefly vortexed, incubated at 37°C for 45 min (200 rpm), then spread on synthetic drop-out (DO) media (Formedium), and incubated at 30°C. Non-transformed cells were grown on yeast-extract peptone dextrose (YPD). G418 (Sigma-Aldrich) was added to media used for the growth of mutant strains. For hygromycin treatments, cells were grown overnight in liquid DO media and diluted to an OD_600_ of 1.0. A serial dilution of each culture was then spotted onto plates containing DO media with added hygromycin B (TOKU-E) (50–75 *µ*g mL^−1^).

### Reverse transcription PCR (RT-PCR) and reverse transcription quantitative PCR (RT-qPCR)

RNA was extracted from snap-frozen samples using an RNeasy minikit (Qiagen) and analyzed by NanoDrop 1,000 spectrophotometer (Thermo Fisher Scientific). Approximately 1.5 *µ*g of RNA was treated with RQ1 DNase (Promega) and cDNA was synthesized using oligo(dT) primers and SuperScript II Reverse Transcriptase (Thermo Fisher Scientific). For semi-quantitative RT-PCR, synthesized cDNA was used in PCR reactions specific to the gene of interest and *ACTIN7*. RT-qPCR was performed on 45 ng template cDNA using Brilliant III Ultra-Fast SYBR Green QPCR Master Mix with Low ROX (Agilent), using an AriaMx Real-Time PCR System (Agilent). Relative expression was calculated using the 2^−ΔΔCT^ method (Livak and Schmittgen 2001) normalized to *ACTIN7.* All primers used are listed in Supplemental Table S1.

### Phylogenetic tree construction

Putative DOA10-like protein sequences were identified by BLASTP at NCBI using *Sc*DOA10 as the template sequence. Sequences were aligned in SEAVIEW5 using the Clustal O method, and the phylogenetic tree was constructed using SEAVIEW5 and BIONJ (BIO Neighbor-Joining) method (Gouy et al. 2021), where Poisson zorrection and bootstrap testing is performed with 1,000 iterations.

### Histochemical staining

*Arabidopsis* seedlings were incubated in GUS stain solution (100 mM phosphate buffer (pH 7.0), 2 mM X-gluc (X-GLUC Direct) 0.1% v/v Triton-X-100, 1 mM potassium ferricyanide, 1 mM potassium ferrocyanide) at 37°C for 24 h. Seedlings were cleared and fixed in 3:1 ethanol/acetic acid, mounted onto microscope slides in 50% v/v glycerol, and imaged on a dissecting microscope. At least three biological repeats were conducted for each transgenic line.

### Cell fractionation

Cell fractionation was performed based on Abas and Luschnig (2010). The tissue was lysed by grinding in extraction buffer (100 mM Tris-HCl (pH 8.0), 5% v/v glycerol, 10 mM EDTA, 10 mM EGTA, 5 mM KCl, 1 mM DTT), supplemented with cOmplete, Mini, EDTA-free Protease Inhibitor Cocktail (Roche), and precleared by centrifugation at 630 × g (10 min). High-speed centrifugation (21,000 × g) was then carried out for 2 h (4°C) to pellet the microsomal fraction. The supernatant (soluble fraction) was removed for analysis, and the pellet was washed with 150 *μ*L of wash buffer (100 mM Tris-HCl (pH 8.0), 5 mM EDTA, 150 mM NaCl), and resuspended in buffer (10 mM Tris-HCl [pH 8.0], 0.5 mM EDTA, 150 mM NaCl). A 1/5th volume of 5 × sample buffer (300 mM Tris-HCl [pH 6.8], 50% v/v glycerol, 25% β-mercaptoethanol, 10% SDS, 0.05% bromophenol blue) was added for analysis by SDS-PAGE and immunoblotting. Fractionation experiments were conducted at least twice for each transgenic line.

### Confocal microscopy

The *AtDOA10A* CDS (pDONR221) was cloned into pB7WGY2.0 to produce *35S::eYFP-AtDOA10A* and transiently co-expressed in *Nicotiana benthamiana* epidermal cells with the ER marker VMA12-RFP (Viotti et al. 2013) via *A. tumefaciens*-mediated transformation (Kong et al. 2015). After 72 h, leaves were additionally infiltrated with 2 *µ*g µL^−1^ 4′,6-diamidin-2-phenylindol (DAPI, Sigma-Aldrich) in ddH_2_O supplemented with 1:20,000 v/v Triton-X (100%). Fluorescence was analyzed by confocal laser scanning microscopy using a Nikon-automated Ti-inverted microscope equipped with a Yokogawa CSU-X1 confocal scanning unit, a Hamamatsu C9100-02 EMCCD camera, and a Nikon S Fluor 40× numerical aperture 1.3 oil-immersion objective (Nikon). Images were taken in five channels (RFP, 561/615; DAPI, 405/445 nm; EYFP, 488/527 nm; autofluorescence, 485/705 nm; and brightfield) and processed with Fiji image analysis software.

### RNA-sequencing

RNA was extracted from biological triplicates of pooled 10-day-old seedlings grown vertically on ½ MS plates, and samples were sequenced and analyzed by Novogene UK. Briefly, mRNA was purified from total RNA using poly-T oligo-attached magnetic beads. After fragmentation, the first-strand cDNA was synthesized using random hexamer primers followed by the second-strand cDNA synthesis. The library was ready after end repair, A-tailing, adapter ligation, size selection, amplification, and purification. The library was checked with qubit and real-time RT-qPCR for quantification and bioanalyzer for size distribution detection, before pooling and sequencing on Illumina platforms to generate >20 M pair-end clean reads. Sequencing quality control, mapping, quantification, and differential gene expression analysis were carried out using HISAT2 software and RPKM calculations for each gene, and DESeq2 and edgeR packages in R were used to generate lists with DEGs as described. GO enrichment analysis was carried out at geneontology.org (Ashburner et al. 2000; Mi et al. 2019).

### Nt-acetylome profiling

Three biological replicates of 10-day-old *Arabidopsis* Col-0 WT and *AtDOA10a/b* RNAi 4-2 seedlings (∼400 mg for each sample) were processed following exactly the previously described SILProNAQ protocol (Bienvenut et al. 2017a). Following protein extraction and Bradford assay, 1 mg of proteins was denatured and then labeled on their N-termini and lysine ε-amino groups using N-acetoxy-[^2^H_3_]succinimide (25 *µ*mol/mg). The labeled proteins were subjected to trypsin digestion (100 U/mg protein), and the resulting peptide mixtures were fractionated on a Strong Cation eXchange (SCX) chromatography column (Polysulfoethyl A, 200 × 2.1 mm, 5 *µ*m, Hichrom, UK) to separate the acetylated N-termini and the non-acetylated internal peptides. N-termini-enriched fractions (fractions 2 to 11) were individually analyzed by LC-MS/MS on an LTQ Orbitrap Velos mass spectrometer. Raw data files were processed by Mascot Distiller, using the latest Araport11 database for identification, and with mass tolerance settings of 10 ppm and 0.5 Da for the parent and fragments ions, respectively. Quantification results were exported and then parsed by the EnCOUNTer script (Bienvenut et al. 2017b) to obtain the data sets. Manual consolidation was then performed on all samples. This included combining the biological replicates of each condition, averaging their NTA levels and the corresponding standard deviations, as well as calculating ratios and *P*-values (two-tailed *t*-test).

### Cycloheximide chases and protein extractions

Yeast cycloheximide (CHX) chases were performed according to the protocol described by (Buchanan et al. 2016). Transformed colonies were grown overnight in liquid DO media at 30°C before subculturing into 30 mL fresh media to an OD_600_ of 0.2. The secondary cultures were then grown at 30°C to an OD_600_ of 1.0, then 8 mL of culture was pelleted by centrifugation before resuspending in 3.2 mL fresh 30°C DO media with 250 *µ*g mL^−1^ CHX (in DMSO) and briefly vortexing. Approximately 950 *µ*L samples were removed at specified time points, added to 50 *µ*L of ice-cold Stop Mix (1 M NaN_3_, 100 *µ*g mL^−1^ BSA), centrifuged (30 s) at 6500 × g, and snap-frozen. Total protein was extracted from pellets in 50 *µ*L of extraction buffer (0.1 M NaOH, 50 mM EDTA, 2% v/v β-mercaptoethanol, 2% v/v SDS) and heated to 90°C for 20 min, with 0.67 *µ*L 3 M acetic acid added halfway through. Approximately 12.5 *µ*L of sample buffer (250 mM Tris-HCl (pH 6.8), 50% v/v glycerol, 0.05% bromophenol blue) was subsequently added. Cells were then pelleted by centrifugation, and the supernatant was used for analysis.

*Arabidopsis* CHX chases were performed on seedlings grown vertically for 7 days and then transferred to liquid ½ MS for a further 3 days, at which point 300 *µ*g mL^−1^ CHX ± 50 *µ*M bortezomib was added. At specified time points, 30 seedlings were blotted dry and snap-frozen. Total protein was extracted by grinding in lysis buffer (10 mM Tris-HCl (pH 8.0), 150 mM NaCl, 0.5 mM EDTA, 0.1% v/v SDS, 1% v/v Trition-X-100) with added cOmplete, Mini, EDTA-free Protease Inhibitor Cocktail (Roche). Following pelleting of cell debris, 1/5th volume 5 × sample buffer (above) was added to the supernatant prior to SDS-PAGE. All CHX chases were conducted three to four times.

### SDS-PAGE and immunoblotting

SDS-PAGE and immunoblotting were performed using the BIO-RAD Mini-PROTEAN system. Protein concentrations were quantified via Bradford assay, separated on 10% v/v polyacrylamide gels, transferred to a PVDF membrane, and blocked in 5% non-fat milk in TBST. Membranes were probed with primary antibodies: 1:4000 anti-β-actin (Abcam ab184220), 1:5000 anti-β-tubulin (Sigma-Aldrich T8328), 1:2500 anti-AtCNX1/2 (Agrisera AS12 2365), 1:4000 anti-AtUGPase (Agrisera AS05 086), 1:1000–1:3000 anti-GUS (Sigma-Aldrich G5420), 1:1000 anti-GFP/YFP (Roche 11814460001), 1:4000 anti-HA (Sigma-Aldrich H3663), and 1:1000 anti-Myc (Antibodies.com A85281). Horseradish peroxidase-conjugated anti-mouse (Sigma-Aldrich A5278) or anti-rabbit (Cell Signaling Technology 7074) secondary antibodies were subsequently added to allow development with Pierce ECL Western Blotting Substrate (Thermo Fisher Scientific) and ECL Hyperfilm film (Amersham). Band densities were quantified relative to the most intense band following normalization to actin/tubulin using Fiji image analysis software. All stability assays were conducted at least three times.

### Accession numbers

RNA-seq data are available at the NCBI GEO database with accession codes GSE236282 (for *doa10a/b RNAi 4-2* and its related Col-0 WT, *amiNAA10*, and *Atnaa20* data sets) and GSE161571 (for Col-0 WT data sets related to *amiNAA10* and *Atnaa20* analyses). Nt-acetylome data are available at the Proteomics Identification Database (PRIDE) with accession code PXD043217.

## Supplementary Material

kiad406_Supplementary_Data

## Data Availability

All data are provided along with the manuscript and its Supplemental material.

## Supplemental data

The following materials are available in the online version of this article.

**Supplemental Figure S1.** Full-length amino acid residue sequence alignment of ScDOA10, AtDOA10A, and AtDOA10B.

**Supplemental Figure S2.** Transmembrane and subcellular localization predictions for yeast and *Arabidopsis* DOA10 and SQE proteins.

**Supplemental Figure S3.** *At*DOA10A and B mutant and complementation line characterization.

**Supplemental Figure S4.** Global NTA variation comparisons for plastidic, cytosolic, and nuclear proteins in *Adoa10a/b RNAi 4-2* and Col-0.

**Supplemental Figure S5.** Prevalence of Nt-acetylation in yeast and *Arabidopsis* Nt-peptides with the listed amino acid sequences.

**Supplemental Figure S6.** Expression and CHX chase analysis of AtSQE1-Myc transgenic lines.

**Supplemental Figure S7.** Fold changes of *AtDOA10A* and *AtDOA10B* expression in 6-day-old seedlings following 4-h treatment with tunicamycin, as determined by RT-qPCR, relative to DMSO-treated seedlings.

**Supplemental Table S1.** List of primers used in this study.

**Supplemental Data Set 1.** List of DEGs.

**Supplemental Data Set 2.** Nt-acetylome data.

**Supplemental Data Set 3.** Prevalence of Nt-acetylation in yeast and *Arabidopsis* Nt-peptides in NterDB.
