# Supplementary material for: Nt-acetylation-independent turnover of SQUALENE EPOXIDASE 1 by Arabidopsis DOA10-like E3 ligases
Source: Plant Physiol. 2023 Jul 10;193(3):2086–104. doi: 10.1093/plphys/kiad406 (PMC10602611; doi:10.1093/plphys/kiad406)
Supplement: kiad406_Supplementary_Data [file kiad406_supplementary_data.zip › Supplemental Data.pdf]

```
ScDOA10      1 MDVD----------------SDVNVSR-------------LRDELHKVANEETDTATFND
AtDOA10A     1 MEISPADSLSISGAAASEVVSEPSVSSSSSSSSPNQASPNPFSNMDPAVSTATGSRYVDD
AtDOA10B     1 MEISPAEDKLVG--------------------------------SG---EAVTTEEVSDI

ScDOA10     32 DAPSGATCRICRGEATEDNPLFHPCKCRGSIKYMHESCLLEWVASKNIDISKPGADVKCD
AtDOA10A    61 DEDEEDVCRICRNPGDADNPLRYPCACSGSIKFVHQDCLLQWLNHSN--------ARQCE
AtDOA10B    26 NNKAVDICRICQSPEEPDNPLRHPCACRGSLKYIHSDCLFLWLNRRK--------RNHCE

ScDOA10     92 ICHYPIQFKTIYAENMPEKIPFSLLLSKSILTFFEKARLALTIGLAAVLYIIGVPLVWNM
AtDOA10A   113 VCKHPFSFSPVYADNAPSRLPFQEFVVGIAMKACHVLQFFLRLSFVLSVWLLTIPFITFW
AtDOA10B    78 ICKRSYSIVPVYSENAPERLPWHEFLMGLLMRALRFMNL-------ILPWILMMPFNAYC

ScDOA10    152 FGKLYTMMLDGSSPYPGDFLKSLIYGYDQSATPELTTRAIFYQLLQNHSFTSLQFIMIVI
AtDOA10A   173 IWRLAF-----VRT----------FGEAQR---------LFLSHI------STTVILTDC
AtDOA10B   131 FSF---------RP----------WGRESE---------FVNQTV---------FELSLR

ScDOA10    212 LHIALYFQYDMIVREDVFSKMVFHKIGPRLSPKDLKSRLKERFPMMDDRMVEYLAREMRA
AtDOA10A   203 LHGFLLSA-----------SIVFI----FLGAT---------------SLRDYF-RHLRE
AtDOA10B   154 FPGLFYTA-----------QIVSSATEMVVQME---------------TIRVLL-RR---

ScDOA10    272 HDENRQEQGHDRLNMPAAAADNNNNVINPRNDNVPPQDPNDHRNFENLRHVDELDHDEAT
AtDOA10A   232 LG--GQEERDDDVDR---------------NGA---------------------------
AtDOA10B   184 ------------------------------------------------------------

ScDOA10    332 EEHENNDSDNSLPSGDDSSRILP-GSSSDNEEDEEAEGQQQQQQPEEEADYRDHIEPNPI
AtDOA10A   248 -------RAARRPAGQANRNLAGEGNGEDAGDQGAAVGQIARRNPENVLA---RLD-IQA
AtDOA10B   184 -----------HPE--FLRRMIILENGLKDRDVTGIVLLLA--N---------HLQ-ILC

ScDOA10    391 DMWANRRAQNEFDDLIAAQQNAINRPNAPVFIPPPAQNRAGNVDQDEQDFGAAVGVPPAQ
AtDOA10A   297 ARLEAQV--------------------EQMFD--GLDDADGAEDVPFDELVGM-------
AtDOA10B   219 DWWHDQLLQLPF---------------LHIFQRGPLALAFVPRNTPLHQFGAI-------

ScDOA10    451 ANPDDQGQGPLV--INLKLKLLNVIAYFIIAVVFTAI------YLAISYLFPTFIGFG--
AtDOA10A   328 -------QGPVFHLVENAFTVLASNMIFLGVVIFVPFTLGRIILYHVSWLFAAARGPAVA
AtDOA10B   257 -------RRVFSLLSDNTFAVLAINIYWSFFRVLLPFSIGRVVLVLLRCLPH--------

ScDOA10    501 -----------LLKIYFGIF-------------KVILRGLCHLYYLSGAHI-----AYNG
AtDOA10A   381 ASLHLTDTGLSLENITLKSALTAVSNLTSEGQGNGLLGQLTEMMKVNGSELNGANNTLSV
AtDOA10B   302 --------GWIAE---------------------------------NASE----------

ScDOA10    532 LTKLVPKVDVAMSW--------ISDHLIHDIIYLYNGYTENTMKHSIFIRALPALTTYLT
AtDOA10A   441 ATDLLKGSTVGASKLSDITTLAVGYMFIVFLVFLYLGIIA--------------LIRYAK
AtDOA10B   311 --------------------MAAGDMVIRSVLLACLG-----------------------

ScDOA10    584 SVSIVCASSNLVSRGYGRENGM--SNPTRRLIFQILFALKCTFKVFTLFFIELAGFPILA
AtDOA10A   487 ------GEPLTVGRFYGIASIVEAVPSLLRQFLAAMRHLMTMIKVAFLLVIELGVFPLMC
AtDOA10B   328 -------G---------------VFTMSRDTYLTSVRTFLPSVKDTFILSFKLGVLPWLL

ScDOA10    642 GVMLDFSLFCPILASNSRMLWVPSICAIWPPFSLFVYWTIGTLYMYWFAKYIGMIRKNII
AtDOA10A   541 GWWLDVCTVRM---FGKTMSHRVQFLSISPLASSLVHWVVGIMYMLQISIFVSLLR-GVL
AtDOA10B   366 GCWLHFCTFPI---LGKTASHTVEVLSDYPLMA-DKHWLMGTLYLVSALSCMELIQ-KIV

ScDOA10    702 RPGVLFFIRSPEDPNIKILHDSLIHPMSIQLSRLCLSMFIYAIFIVLGFGFHTR------
AtDOA10A   597 RPGVLYFLRDPADPNYNPFRDLIDDPVHKHARRVLLSVAVYGSLIVMLVFLPVKLAIRMA
AtDOA10B   421 QKRALWYLLDVAEPNYKVTK--------LHLGPILLAFALHGTMVVIVLHLPIKTISLIS
```

```
ScDOA10    756  -IFFPFMLKSNLLSVPEAYKPTSIISWKFNTILLTLYFTKRI--LESSSYVKPLLERYWK
AtDOA10A   657  PSIFPLDIS---VSDPFTEIPADMLLFQIC---I-PFIIEH---FRLRTTIKSLLRCW--
AtDOA10B   473  QSFFPLQFG---VYEDE--FVFGLLVAYMC---L-VIFGPRWLANLIRPSIRPIVHKW--

ScDOA10    813  TIFKLCSRKLRLSSFILGKDTPTERGHIVYRNLFYKYIAAKNAEWSNQELFTKPKTLEQA
AtDOA10A   705  --FTGVGWALGLTDFLLPRPEDNIGQDN---------------------GNGEPGRQNR-
AtDOA10B   522  --VITISSLLKLSDFLLGEPRKHRANHN---------------------M--------R-

ScDOA10    873  EELFGQVRDVHAYFVPDGVLMRVPSSDIVSRNYVQTMFVPVTKDDKLLKPLDLERIKERN
AtDOA10A   741  -------AQVLQVGGPDRAMAALPVADDPNRS----------------------RLRAGN
AtDOA10B   550  -------LRCLVFGIAEGSMVSLHGS----------------------------------

ScDOA10    933  KRAAGEFGYLDEQNTEYDQYYIVYVPPDFRLRYMTLLGLVWLFASILMLGVTFISQALIN
AtDOA10A   772  VNTGEEYEDDDEQSDSDRYN--------FVVRIILLLLVAWVT--LLLFNSALIVVPVSL
AtDOA10B   569  -QSDTTCEK--DTNEQRDKR--------FMVRIGVMLILASLS--MFLVSTTFMALPILV

ScDOA10    993  FVCSFGFLPVVKLLLGER-NKVYVA-------WKELSDISYSYLNIYYVCVGSVCLSKIA
AtDOA10A   822  GRALFSAIPILPITHGIKCNDLYAFVIGTYAFWTTISGARYAI-----------------
AtDOA10B   616  GRAFFHSISFFMLSFGLKHDDLCAFWIGFCILRGIYIITCFVY-----------------

ScDOA10   1045  KDILHFTEGQNTLDEHAVDENEVEEVEHDIPERDINNAPVNNINNVEEGQGIFMAIFNSI
AtDOA10A   865  ---EHVKSKRTSV------------------------------------------LLNQI
AtDOA10B   659  ---DHFITGRTDL------------------------------------------LLNHV

ScDOA10   1105  FDSMLVKYNLMVFIAIMIAVIRTMVSWVVLTDGILACYNYLTIRVFGNSSYTIGNSKWFK
AtDOA10A   880  W----------KWCGIVFKSSVLLAIWVFIIPVLIGLLFELLV--IVPMRVPVDESPVFL
AtDOA10B   674  L----------MF----IRNVLLFSIWISVIPGVLGLLIDLMI--IIPSQVPLGESPVYN

ScDOA10   1165  -YD----ESLLFVVWIISSMVNFGTGY-----KSLKLFFRNRNTSKLNFLKTMALELFKQ
AtDOA10A   928  LYQDWALGLIFLKIWTRLVMLDHMLPIVDDSWRAKFERVREDGFSRLQGLWV-----LR-
AtDOA10B   718  LLHDWLIGVVVLHIWIFLTMLTRINCFATVAWREKLQRIRSVTINRLPFTWL-----IR-

ScDOA10   1215  GFLHMVIYVLPIIILSLVFLRD---VSTKQIIDISHGSRSFTLSLNESFPTWTRMQDIYF
AtDOA10A   982  ------EIVFPIV-MKLLTALCVPYVLARGVFP----MLGYPLVVNSAVY--------RF
AtDOA10B   772  ------DVIGSII-VSLLFTLCVPYVVVNSLFP----ILGFSSAVNLTVQ--------RF

ScDOA10   1272  GLLIALESFTFFFQATVLFIQWFKSTVQNVKDEVYTKGRALENLPDES------------
AtDOA10A  1023  -AWIGCLSVSLFCFCAKRCHVWFRNLHNSIRDDRYLIGRRLHNFGEAALANQNQNQSSED
AtDOA10B   813  -IWPAILALIPIWFSVKLIRDLILYLHKLEFDNRYKVGERLVDFTEDLE-----------

ScDOA10         ---------------------------
AtDOA10A  1082  AGDGVLIGREGDVDNGLRLRRAIQQEA
AtDOA10B        ---------------------------
```

Supplemental Figure S1. Full length amino acid residue sequence alignment of ScDOA10, AtDOA10A and AtDOA10B. Blue bar denotes RING-CH domain, green bar conserved TD region. Sequences were obtained from NCBI and aligned using clustalW2 (now Clustal Omega: https://www.ebi.ac.uk/Tools/msa/clustalo/), then converted to the output shown using Boxshade 3.2 (https://embnet.vital-it.ch/software/ BOX_form.html), with the fraction of sequences that must agree for shading set to >0.5.

a

Prediction Model	Predicted number of Transmembrane domains		
	ScDoa10	AtDOA10A	AtDOA10B
TMHMM 2.0	14	14	13
TMpred	14	16	14
Phobius	16	16	14

b

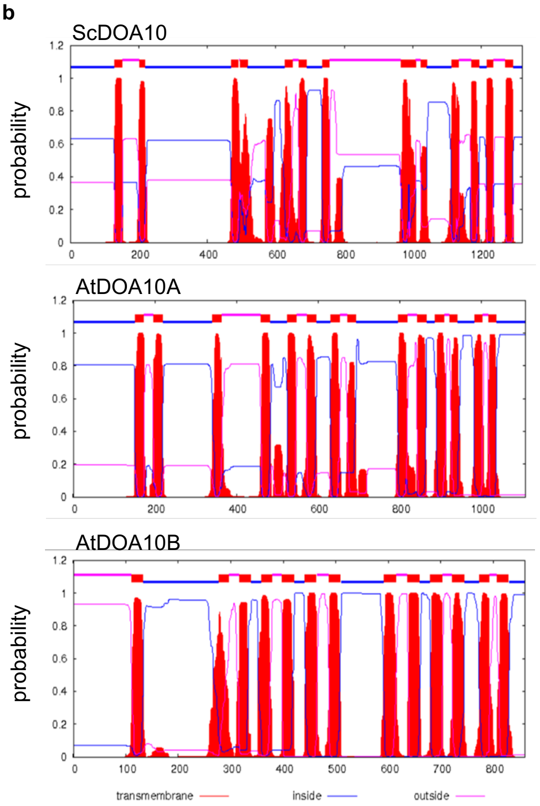

c

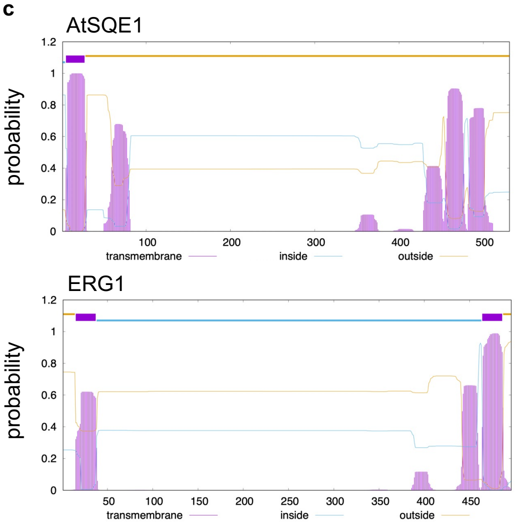

d

Protein	Prediction
*Sc*DOA10	ER
*At*DOA10A	ER
*At*DOA10B	ER
ERG1	ER
*At*SQE1	ER

Supplemental Figure S2. Transmembrane and subcellular localization predictions for yeast and Arabidopsis DOA10 and SQE proteins. (**A**) Estimated number of TM domains in *At*DOA10A and B relative to *Sc*DOA10 using the three different prediction models shown. (**B**) TMHMM 2.0 predicted transmembrane topologies for *Sc* and *At*DOA10s. (**C**) TMHMM 2.0 predicted transmembrane topologies for AtSQE1 and ERG1. (**D**) Subcellular localization predictions for DOA10 and SQE proteins using Euk-mPLoC 2.0 (Chou and Shen 2010), which predicts eukaryotic protein localizations independent of the organism of origin.

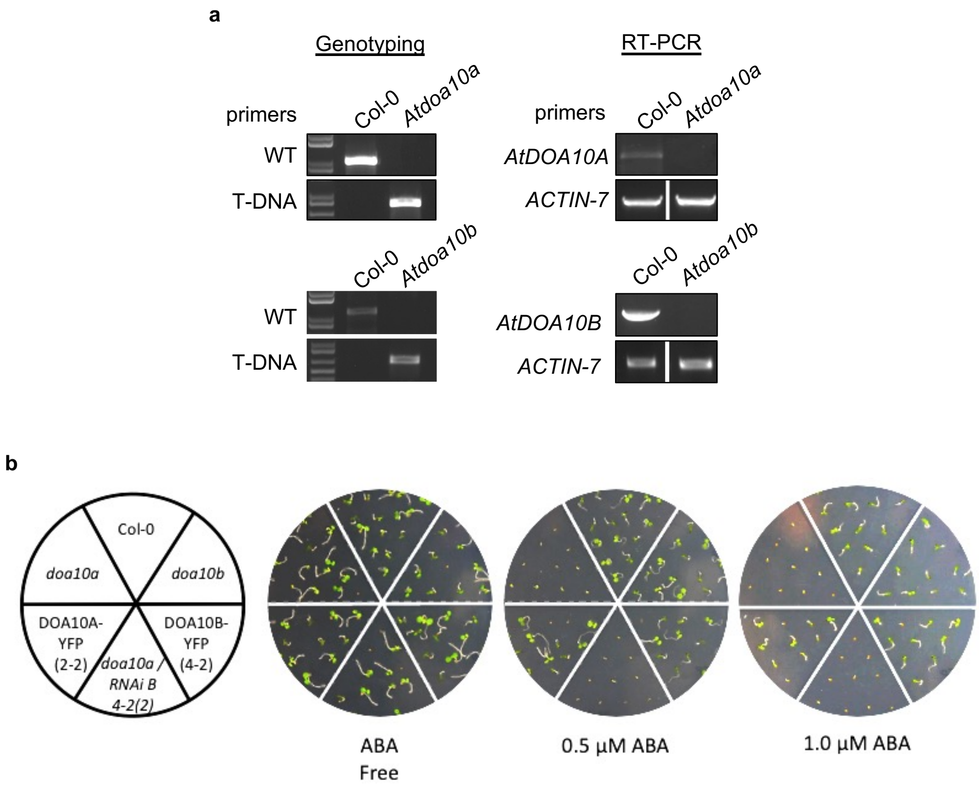

Supplemental Figure S3. *At*DOA10A and B mutant and complementation line characterization. (**A**) Genotyping PCR and RT-PCR confirming homozygous T-DNA insertion and absence of full-length mRNA expression in *Atdoa10a* and *Atdoa10b* T-DNA insertion lines. (**B**) seedling ABA-sensitivity assays showing ABA-hypersensitivity in *Atdoa10a* is reverted by complementation with *pDOA10A::AtDOA10A-YFP* (line 2-2).

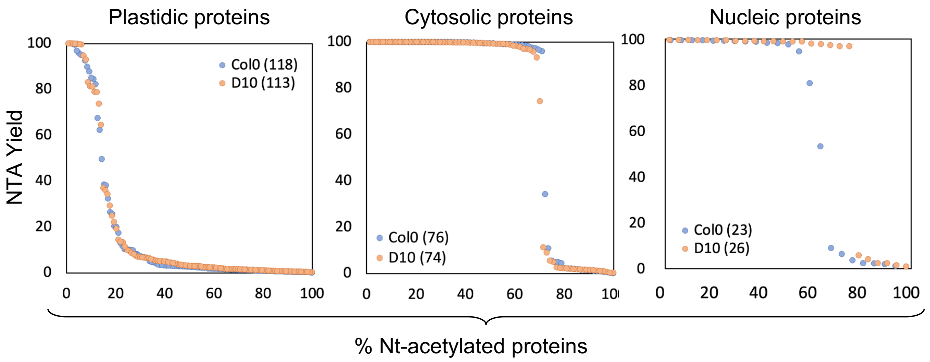

Supplemental Figure S4. Global NTA variation comparisons for plastidic, cytosolic and nuclear proteins in *Adoa10a/b RNAi 4-2* and Col-0. For each sample, the peptides were sorted in decreasing order of %NTA (quantitated only). Each N-ter entry was assigned a number corresponding to its relative position. These proteins numbers are plotted with matching %NTA for all acetylation positions. For nuclear N-termini the the low number of retrieved peptides introduces bias; looking at each individual N-terminus revealed no variation.

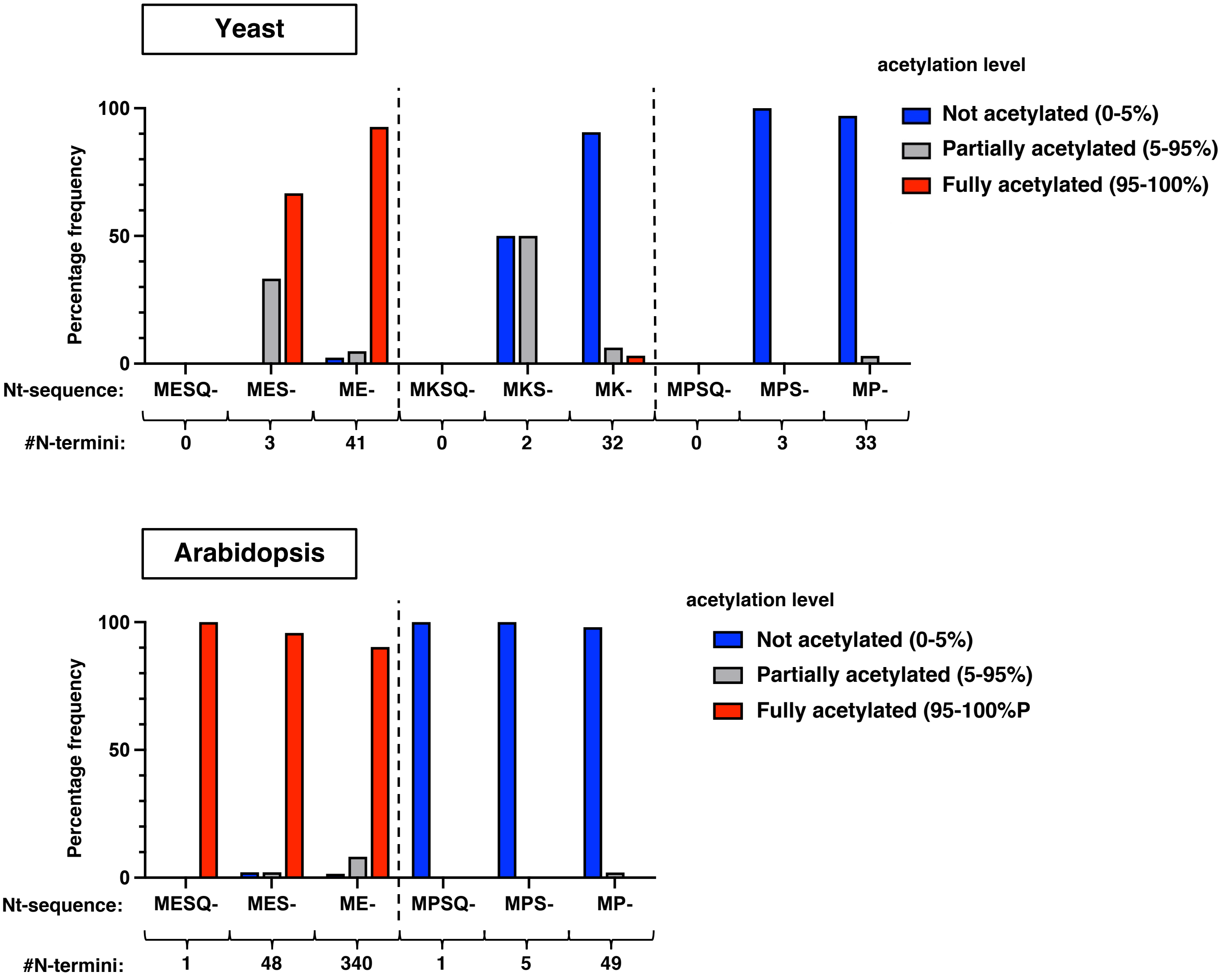

Supplemental Figure S5. Prevalence of Nt-acetylation in yeast and Arabidopsis Nt-peptides with the listed amino acid sequences. Data were compiled from the N-terminomics database NterDB (https://nterdb.i2bc.paris-saclay.fr/). The number of peptides for which Nt-acetylation has been quantified is given below the y-axis. Percentage frequency corresponds to the proportion of these peptides within each of the listed Nt-acetylation level categories: Not acetylated (0-5%), partially acetylated (5-95%) and fully acetylated (95-100%). For example, the single Arabidopsis MESQ Nt-peptide (matching ME-AtSQE1) for which data is available (AT5G38650) is 97.73% Nt-acetylated and therefore Arabidopsis MESQ peptides are shown as 100% fully acetylated. The single Arabidopsis MPSQ Nt-peptide (matching MP-AtSQE1) for which data is available (AT5G06830) is 0.69% Nt-acetylated and is therefore shown as 100% not acetylated. See also Supplemental Table 3.

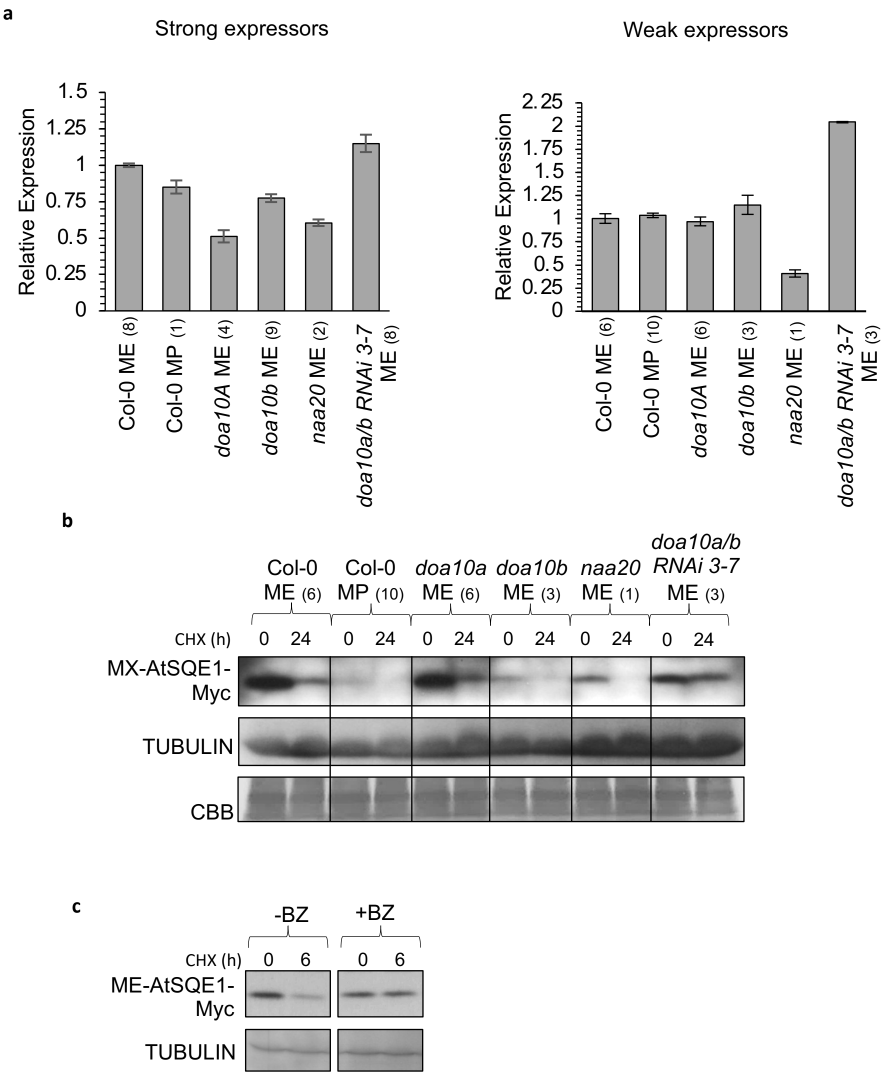

Supplemental Figure S6. Expression and CHX-chase analysis of AtSQE1-Myc transgenic lines. (**A**) RT-qPCR of *35S::ME/MP-AtSQE-Myc* mRNA levels in the lines shown. Two independent lines for each transgene/genotype combination were analyzed and divided into "strong expressors" and "weak expressors". "Strong expressors" were analyzed further in Figure 7. Relative expression levels were calculated through normalisation to *AtACT7* and are the average of three biological repeats. (**B**) CHX chase of WT ME- and mutant MP-AtSQE1-Myc "weak expressors" in WT Col-0 and different mutant backgrounds. Tubulin and coomassie brilliant blue (CBB) loading controls are shown. (**C**) CHX chase of WT ME-AtSQE1-Myc in Col-0 +/- Bortezomib (BZ).

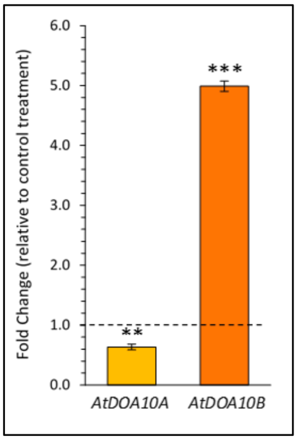

Supplemental Figure S7. Fold changes of *AtDOA10A* and *AtDOA10B* expression in 6-day old seedlings following 4-hour treatment with tunicamycin, as determined by qRT-PCR, relative to DMSO-treated seedlings. Fold changes were calculated according to the delta delta Ct method and data presented are the averages of 3 biological repeats (±SE). Statistical significance was determined using a student's t- test (** $p<0.01$; *** $p<0.001$).

Supplemental Table S1. List of primers used in this study.

**Genotyping (Arabidopsis)**		
doa10a_LP	AT4G34100	AATTTCTCCTCTGGCAAGCTC
doa10a_RP	AT4G34100	AAGAGTCACCCATGCAACAAG
doa10a_BP	AT4G34100	GGGCTACACTGAATTGGTAGCTC
doa10b_LP	AT4G32670	GGGGTGTCTCCTTAAAAGCAC
doa10b_RP	AT4G32670	TTTCCTTTTCTTTTGCGTGAG
doa10b_BP	AT4G32670	TGGTTCACGTAGTGGGCCATCG
DOA10_RT-PCR_Exon3_F	AT4G34100	CTCTGGCAAGCTCACTTGTTC
DOA10A_RT-PCR_Exon8_R	AT4G34100	GGCACGCATAAAGCCGTTAG
DOA10B_RT-PCR_Cloning_F	AT4G32670	CACCATGGAGATTTCTCCGGCG
DOA10B_RT-PCR_Cloning_RO	AT4G32670	CTCGAGATCTTCAGTGAAATCG
ACT7_F	AT5G09810	ATGGCCGATGGTGAGGATAT
ACT7_R	AT5G09810	GAGCACAATACCGGTTGTACG
**DOA10A/B Cloning**		
DOA10A_Cloning_F(pDNR)	AT4G34100	GGGGACAAGTTTGTACAAAAAAGCAGGCTGCA TGGAGATTTCCCCGGCCGATTC
gDOA10A_Cloning_F(pDNR)	AT4G34100	GGGGACAAGTTTGTACAAAAAAGCAGGCTTCG CATAATATACAAAAGGTCGTCC
DOA10A_Cloning_RO(pDNR)	AT4G34100	GGGGACCACTTTGTACAAGAAAGCTGGGTCAG CTTCTTGTTGGATTGCAC
DOA10A_Cloning_RC(pDNR)	AT4G34100	GGGGACCACTTTGTACAAGAAAGCTGGGTCCT AAGCTTCTTGTTGGATTGCAC
DOA10B_Cloning_F(TOPO)	AT4G32670	CACCATGGAGATTTCTCCGGCG
gDOA10B_Cloning_F(TOPO)	AT4G32670	CACCAAAGCTCAAGAAATCGGTACTC
DOA10B_Cloning_RO(TOPO)	AT4G32670	CTCGAGATCTTCAGTGAAATCG
SQE1_Cloning_F(TOPO)	AT1G58440	CACCATGGAGTCACAATTATGGAATTGG
SQE1_Cloning_RO(TOPO)	AT1G58440	TGAACATTTGGTTTCTCCAACTG
**Confirmation of transgene expression (yeast)**		
DOA10A_Cloning_F(TOPO)	AT4G34100	CACCATGGAGATTTCCCCGGC
DOA10A_Exon1_R	AT4G34100	GCACAAAACTCAACCGCAGA
DOA10B_Cloning_F(TOPO)	AT4G32670	CACCATGGAGATTTCTCCGGCG
DOA10B _Exon1_R	AT4G32670	TGCAAATCTCGCAATGGTTG
DOA10A_Exon5_F	AT4G34100	TCCGTGCTGGGAATGTCA
DOA10B_Exon6_F	AT4G32670	TATGTTTCTTGTTAGTACAACCTTCATG
eG/YFP_R		GCTGAACTTGTGGCCGTTTA
ScUBC6_F	YER100W	GATACTTGGAATCCTGGCTGGTCTGTCTC
ScUBC6_R	YER100W	AAAGGGTCTTCTGTTTCATCACCTGTATTTGC
**Confirmation of transgene expression (Arabidopsis)**		
SQE1_Cloning_F(TOPO)	AT1G58440	CACCATGGAGTCACAATTATGGAATTGG
HAtag_R	AT1G58440	ATAGGATCCTGCATAGTCCG
SQE1-Myc_qPCR_F	AT1G58440	GCGGAAGGAGTTAGGCAGAT
SQE1-Myc_qPCR_R	AT1G58440	ACCCGCTGTTATCAACCACTT
**Tissue-specific / RNAi line expression analysis (qRT-PCR)**		
DOA10A_qPCR_Exon8_F	AT4G34100	AATCTGGACTAGACTGGTAATGCT
DOA10A_qPCR_Exon8_R	AT4G34100	GGCACGCATAAAGCCGTTAG
DOA10B_qPCR_Exon1_F	AT4G32670	GGCGGAAGACAAACTCGTTG
DOA10B_qPCR_Exon1_R	AT4G32670	TGCAAATCTCGCAATGGTTG
DOA10B_qPCR_Exon7_F	AT4G32670	GGACTCAAACACGACGATCT
DOA10B_qPCR_Exon7_R	AT4G32670	AGCAAATCGGTTCTTCCCGTT
ACT7_qPCR_F	AT5G09810	CTGGAATGGTGAAGGCTGGT
ACT7_qPCR_R	AT5G09810	GTGCCTAGGACGACCAACAA
**Generation of RNAi lines**		
DOA10B_rnai3_cloF	AT4G32670	CACCGGAGACACCCCGAGTTCTTG
DOA10B_rnai3_cloR	AT4G32670	CAATCCAACCGTGTGGGAGA
DOA10B_rnai4_cloF	AT4G32670	CACCCCGGCGAAGCTGTTACTACA
DOA10B_rnai4_cloR	AT4G32670	TGGGAGACAGCGTAGGAGAA
